# Divergent molecular strategies drive evolutionary adaptation to competitive fitness in biofilm formation

**DOI:** 10.1093/ismejo/wrae135

**Published:** 2024-07-25

**Authors:** Mingxing Tang, Ruixue Yang, Zilin Zhuang, Shuhong Han, Yunke Sun, Peiyu Li, Kewei Fan, Zhao Cai, Qiong Yang, Zhijian Yu, Liang Yang, Shuo Li

**Affiliations:** Department of Otorhinolaryngology, Shenzhen Nanshan People’s Hospital, Shenzhen 518052, China; Community Health Service Center of Southern University of Science and Technology, Nanshan Medical Group Headquarters, Shenzhen 518055, China; Department of Pharmacology, School of Medicine, Southern University of Science and Technology, Shenzhen 518055, China; Department of Pharmacology, School of Medicine, Southern University of Science and Technology, Shenzhen 518055, China; Department of Pharmacology, School of Medicine, Southern University of Science and Technology, Shenzhen 518055, China; Department of Infectious Diseases, Shenzhen Nanshan People’s Hospital, Shenzhen University School of Medicine, Shenzhen 518052, China; Department of Infectious Diseases, Shenzhen Nanshan People’s Hospital, Shenzhen University School of Medicine, Shenzhen 518052, China; Department of Research and Development, Shenzhen Mindray Bio-Medical Electronics Co, Ltd, Shenzhen 518057, China; Department of Otorhinolaryngology, Shenzhen Nanshan People’s Hospital, Shenzhen 518052, China; Department of Infectious Diseases, Shenzhen Nanshan People’s Hospital, Shenzhen University School of Medicine, Shenzhen 518052, China; Department of Pharmacology, School of Medicine, Southern University of Science and Technology, Shenzhen 518055, China; Department of Otorhinolaryngology, Shenzhen Nanshan People’s Hospital, Shenzhen 518052, China; Allergy Prevention and Control Center, Nanshan People’s Hospital, Shenzhen 518052, China

**Keywords:** biofilm evolution, bacteriophage superinfection, c-di-GMP signaling, bacterial virulence, bioinformatic analysis

## Abstract

Biofilm is a group of heterogeneously structured and densely packed bacteria with limited access to nutrients and oxygen. These intrinsic features can allow a mono-species biofilm to diversify into polymorphic subpopulations, determining the overall community’s adaptive capability to changing ecological niches. However, the specific biological functions underlying biofilm diversification and fitness adaptation are poorly demonstrated. Here, we launched and monitored the experimental evolution of *Pseudomonas aeruginosa* biofilms, finding that two divergent molecular trajectories were adopted for adaptation to higher competitive fitness in biofilm formation: one involved hijacking bacteriophage superinfection to aggressively inhibit kin competitors, whereas the other induced a subtle change in cyclic dimeric guanosine monophosphate signaling to gain a positional advantage via enhanced early biofilm adhesion. Bioinformatics analyses implicated that similar evolutionary strategies were prevalent among clinical *P. aeruginosa* strains, indicative of parallelism between natural and experimental evolution. Divergence in the molecular bases illustrated the adaptive values of genomic plasticity for gaining competitive fitness in biofilm formation. Finally, we demonstrated that these fitness-adaptive mutations reduced bacterial virulence. Our findings revealed how the mutations intrinsically generated from the biofilm environment influence the evolution of *P. aeruginosa*.

## Introduction

Biofilms are an assemblage of bacterial cells that are enclosed by self-produced matrixes [1], which constrain bacterial diffusion and simultaneously render protection against physical and chemical disruptions [2]. As a predominant form of bacterial lifestyle, biofilms are often organized either in a surface-attached manner or as non-surface-attached aggregates, where cooperation and competition co-exist [3–6]. Successful biofilm formation and maintenance requires the social cooperation of biofilm-dwelling cells, including the communal production of signaling molecules [7], polymer matrix [1, 8], and nutrient chelators [9, 10]. However, biofilms are also a hotspot of bacterial competition, at both the intra-species and inter-species level [5, 6]. In fact, even a mono-species biofilm could be an incubator of phenotypic and genotypic heterogeneity, yielding diversified subpopulations that can determine the adaptive potential of the overall community to a changing ecological niche [11–14]. Understanding the molecular bases for biofilm evolution and diversification is of great importance, and experimental evolution (EE) coupled with genome sequencing has been powerful to address this issue [15]. Many studies in this direction identified putatively adaptive mutations in global regulatory circuits, such as the bis-(3′-5′)-cyclic dimeric guanosine monophosphate (c-di-GMP) signaling system or quorum sensing (QS), thus providing theoretical explanations for bacterial adaptation to competitive fitness in a biofilm environment [15–17]. How the downstream physiological functions are recruited to achieve the evolutionary adaptation, however, is poorly understood.

*Pseudomonas aeruginosa* is a model microorganism typically used in biofilm studies [3]. Beyond this, it is also a ubiquitous Gram-negative pathogen that causes many severe acute and chronic infections [18]. Due to the intrinsic resistance to antibiotics, biofilm formation makes *P. aeruginosa* a particularly challenging pathogen in immuno-compromised patients, especially for those with cystic fibrosis [19, 20]. Formation and maintenance of *P. aeruginosa* biofilm is tightly governed by c-di-GMP, a critical secondary messenger synthesized by diguanylate cyclases (DGCs) and hydrolyzed by phosphodiesterases (PDEs) [21–23]. Generally, high levels of c-di-GMP repress swimming motility but promote the production of biofilm matrixes that include exopolysaccharides (EPS), extracellular DNA (eDNA), and proteinaceous adhesins [22, 24]. *Pseudomonas* filamentous (Pf) bacteriophage was recently identified as an important biofilm matrix component [25–27]. As for the *P. aeruginosa* reference strain PAO1, Pf4 exists in multiple forms that all intimately associate with the host bacterium, either as a prophage integrated within the bacterial chromosome, or a circular replicating form within individual PAO1 cells, or as a linear single-stranded DNA (ssDNA) phage [26, 28, 29]. Pf4 encodes a pair of adjacent but bidirectional regulators XisF4 and Pf4r, which determine the Pf4 lifestyle shift between lysogeny and lysis. XisF4 mediates Pf4 excision and replication, yet the repressor *C* protein Pf4r inhibits *xisF4* to prevent excessive Pf4 production [28]. Mature Pf4 virions interact with a spectrum of host or bacterium-derived biopolymers to assemble into highly structured liquid crystals that enhance biofilm adhesion and robustness [25]. Previous works indicated that mutations in Pf4 and c-di-GMP-associated *dgc/pde* genes were often positively selected during *P. aeruginosa* biofilm evolution [16, 17, 30], whereas the specific molecular bases and physiological functions remained lesser understood.

In this study, we firstly launched EE of *P. aeruginosa* PAO1 biofilms and observed phenotypic shifts over a short-term evolution. Using genetic, phenotypic, and phylogenetic approaches, we further demonstrated that the evolved clones gained higher competitive fitness in biofilms via two divergent molecular strategies. These evolutionary strategies were also prevalent among clinical *P. aeruginosa* isolates, and they appeared to reduce bacterial virulence. This study showed that the biofilm features per se drove *P. aeruginosa* evolution.

## Material and methods

### Bacterial strains, plasmids, and growth conditions

Bacterial strains, plasmids, and primers are listed in Supplementary Tables S1 and S2, respectively. Unless specified otherwise, *Escherichia coli* and *P. aeruginosa* strains were grown in Luria-Bertani (LB) and Tryptone Soya Broth (TSB) at 37°C, respectively. *Pseudomonas aeruginosa* growth was measured by monitoring the optical density at 600 nm (OD_600_) using a Spark multiwell reader (Tecan, Switzerland). Antibiotics were used at the following concentrations: chloramphenicol (6 μg mL^−1^), carbenicillin (200 μg mL^−1^), tetracycline (20 μg mL^−1^), ampicillin (100 μg mL^−1^), gentamicin (60 μg mL^−1^), and kanamycin (50 μg mL^−1^).

### Experimental evolution

The EE of *P. aeruginosa* PAO1 biofilm was conducted as previously described with minor modifications [17, 31]. PAO1 overnight culture was diluted in TSB to ~1 × 10^6^ bacteria per mL and 1 mL of dilutions were seeded into the 24-well microplate (Nest, PRC) containing two autoclaved 5 mm glass beads (Merck KGaA, Germany). The microplate was then statically incubated at 37°C for 24 h. After each cycle of evolution, the liquid culture was removed, and the beads were rinsed with sterile phosphate buffered saline (PBS) to remove loosely attached bacteria. Then, two beads were transferred into new tubes with PBS (1 mL), followed by vortex and sonication to generate the biofilm suspensions. The suspensions were then divided into three parts, with the first part (100 μL) plated onto fresh TSB agar to isolate a random colony, the second part (800 μL) for cryopreservation, and the third part (100 μL) as the seed for a new cycle of evolution. A total of six daily transfers were conducted, such that ~58 generations of evolution occurred based on a generation time of 2.468 h (Supplementary Fig. S1A).

### Genome resequencing

The genomic DNA of bacterial populations were extracted using an AxyPerp Bacterial Genomic DNA Miniprep Kit (Corning, USA) and sequenced on a NovaSeq System (Illumina) and a Nanopore sequencing platforms. Genomic reads of the isolates were analyzed using the CLC Genomics Workbench 20 (Qiagen) using the Resequencing analysis module with default parameters.

### Genetic manipulation

In-frame deletion of target genes in PAO1 was performed using the suicide pK18 plasmid, as previously described [17, 32]. To create *pf4r* or *bifA* complementation plasmids, the target genes with their native promoters were PCR-amplified and ligated into mini-CTX1 vector (linearized with HindIII and BamHI) through Gibson assembly as described previously [33, 34]. To create *pf4r* or *xisF4* overexpression plasmids, the coding sequences of target genes were PCR-amplified and ligated into a pHERD20^T^ vector (linearized with EcoRI and HindIII) [35, 36]. The constructed plasmids were then transformed into *E. coli* SM10 λpir, followed by conjugation into *P. aeruginosa* PAO1 or its derivatives. To construct deletion or single nucleotide polymorphism (SNP) mutants, first-time homologous recombinants were screened by selection for gentamicin-resistance, followed by selection for second-time homologous recombinants on LB agar plates with 20% (w/v) sucrose. Successful genetic complementation in *P. aeruginosa* strains was screened on LB agar plates with tetracycline. *Pseudomonas aeruginosa* strains with pHERD20^T^ vectors were selected on LB agar plates containing carbenicillin. All mutants were confirmed by both PCR amplification and Sanger sequencing.

### Bacterial fluorescence labeling and competition assay

*Pseudomonas aeruginosa* were chromosomally labeled with either green fluorescent protein (GFP) or mCherry using a mini-Tn7 transposon delivery system. The pUC18T-mini-Tn7^T^-Gm was integrated into a neutral site of the PAO1 genome by quadri-parental mating using the helper plasmid pRK600 and pBF13 [37]. The integration event was confirmed by PCR amplification and DNA sequencing. Excision of the Gm resistance gene was performed with the pFLP2 plasmid and selected on LB agar containing 5% (w/v) sucrose.

For the pairwise competition assay, *P. aeruginosa* strains tagged with either GFP or mCherry were inoculated in TSB at equivalent ratios. Then, the mixed bacteria were diluted in TSB to ~1 × 10^5^ per mL and 1 mL of the dilution was seeded into the bead-containing 24-well microplate. The microplate was then incubated at 37°C for 24 h at 100 rpm on an orbital shaker. For enzymatic treatment of mixed biofilms to degrade eDNA, 200 μL of 100 μg mL^−1^ DNase 1 (STEMCELL Technology, Canada) was added to 1 mL of TSB medium upon co-inoculation. After 24 h, the biofilm cells were analyzed by Beckman Cytoflex S flow cytometer (Beckman Coulter, USA) to determine the proportion. The relative fitness was determined by the relative proportion in the mature biofilms normalized by the relative inoculum proportion.

### Statistical analysis

Data were processed and visualized by Graphpad Prism v9.0 (San Diego, CA, USA). The mean values were compared by either two-tailed unpaired *t*-test or one-way ANOVA with Tukey *post hoc* tests using a 95% confidence interval. The survival curves of *Galleria mellonella* larvae were compared by using Log-rank (Mantel-Cox) test.

Methods for Pf4 replicative-form (RF) quantification, RT-qPCR and RNA-seq, proteomic analysis, protease assay, bioinformatics analysis, biofilm biomass and morphology observation, plaque assay, motility assays, Alpha2 model of protein structure, protein expression and purification, electrophoretic mobility shift assay (EMSA), transcriptional reporter assays, pyocyanin measurement, cell culture and cytotoxicity assay, and *in vivo* infection of *G. mellonella* larvae were detailed in Supplementary Materials.

## Results

### Adaptation to competitive fitness emerged rapidly in a short-term EE of *P. aeruginosa* biofilms

**Figure 1:**
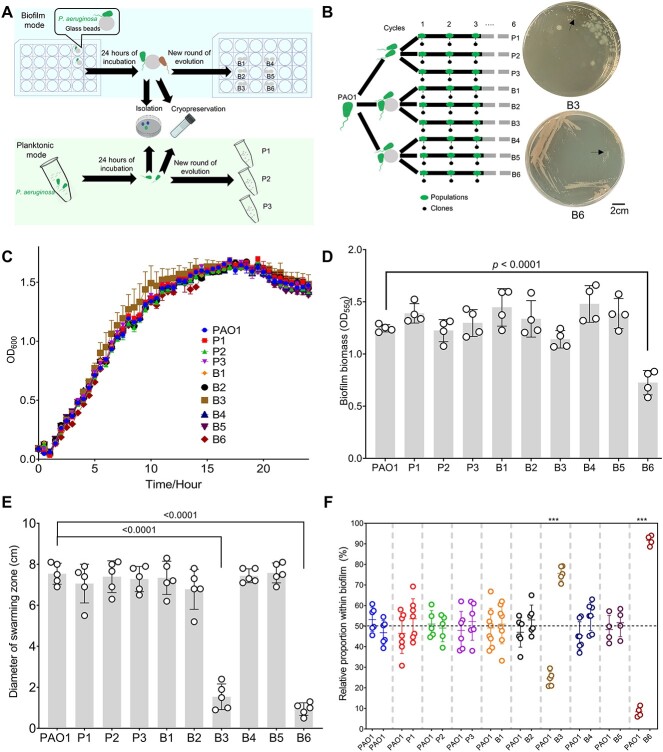
**Phenotypic profiling of the evolved populations after** EE **of *P. aeruginosa* PAO1 biofilms.** (A) Schematic overview of the experimental design used for the evolution study. (B) Six populations were evolved in the biofilm mode whereas another three populations were evolved in the planktonic state as the control. Six serial cycles of evolution were performed. Two evolved populations, B3 and B6, formed small colonies (marked by arrows) on plates. Bar: 2 cm. (C) Growth curves of PAO1 and its derived populations at the 6^th^ cycle of EE. (D) Quantification of biofilm biomass formed by PAO1 and all evolved populations after 24 h of culture in tryptic soy broth (TSB). (E) Quantification of swarming motility of PAO1 and its derived populations after 24 h incubation. (F) Relative fitness of all evolved populations in the comparison with the ancestor PAO1 in biofilms. The percentages of bacteria in each competition assay were normalized by the inoculum ratio. Values correspond to means ± standard deviations. Statistical significance was determined using two-tailed unpaired *t*-test or one-way ANOVA with Tukey *post hoc* tests using a 95% confidence interval. Each experiment was conducted at least three times. ^*^^*^^*^, *P* < 0.001.

We launched EE of *P. aeruginosa* PAO1 biofilms by daily serial passaging of the glass bead surface-attached biofilm community (Fig. 1A), which is a classic *in vitro* model allowing bacteria to undergo the entire biofilm lifecycle of initial adhesion, maturation, dispersion, and re-adhesion in each evolution cycle with an average generation time of 2.468 h. Six replicate biofilm populations (designated as B1, B2, B3, B4, B5, and B6) were thus generated, whereas another three independent planktonic cultures (designated as P1, P2, and P3) were set as the control (Fig. 1A, B). After six cycles, small colony variants (SCVs) formed by two biofilm populations, B3 and B6, emerged on agar plates (Fig. 1B), indicative of biofilm phenotypic shift. We thus analyzed the biofilm-related phenotypes for all the evolved populations at the 6^th^ cycle, in which bacteria had passed through ~58 generations of biofilm growth. Population growth was comparable to that of the ancestor PAO1 (Fig. 1C and Supplementary Fig. S1B). However, despite selection to biofilm communities attached to bead surface, we found most populations, grown either in the planktonic or biofilm mode, did not evolve with higher biofilm production (Fig. 1D). Instead, there was even a drastic decrease in biofilm biomass for the population B6 (Fig. 1D). The swarming motility refers to multicellular flagella-propelled migration on surface and is an important indicator for biofilm formation [38, 39]. Consistently, most evolved populations resembled PAO1 in swarming on semi-solid agar plates, whereas the SCV-producing populations, B3 and B6, were significantly less motile (Fig. 1E). Finally, to determine the competitive fitness, each evolved population was separately subject to competition with the ancestor PAO1 which was tagged with a fluorescence marker. Although most evolved populations were similarly fit, the less motile biofilm populations can significantly outcompete the ancestor (Fig. 1F). Altogether, the phenotypic heterogeneity in biofilm production suggested that divergent molecular strategies are likely opted by B3 and B6 for a convergent adaptation outcome: a higher competitive fitness in biofilms.

### Population genome sequencing to identify mutations adaptive to competitive fitness in biofilms

**Figure 2:**
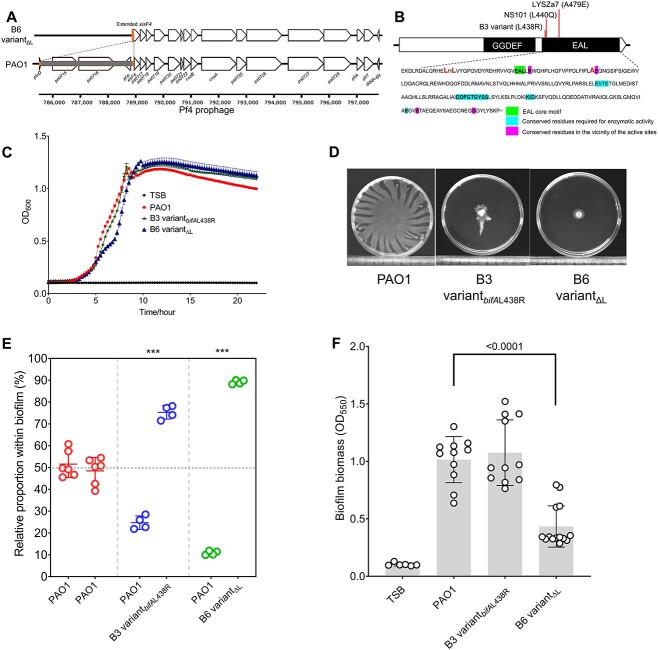
**Two mutations at high frequencies were identified to strongly drive bacterial adaptation to competitive fitness in biofilms**. (A) Genome resequencing revealed a Pf4 prophage region was deleted in the evolved biofilm population B6. The genomic position was shown below the genetic map. (B) Domain structure of PAO1 BifA determined by the Conserved Domains Database in NCBI; the EAL domain sequence is below the predicted structure, with the altered amino acid (in red), core motif, conserved residues in the vicinity of the active sites, and key residues for enzymatic activity marked with different color backgrounds. The mutations detected in the evolved biofilm population B3 and two clinical strains (LYSZa7 and NS101) were pointed out. (C) Growth curve for PAO1 and two isolated derivatives (B3 variant_*bifA*L438R_ and B6 variant_ΔL_) from the evolved biofilm populations at the 6^th^ cycle of EE. (D) Swarming motility of PAO1 and its evolved derivatives after 24 h incubation. (E) Relative fitness of two evolved derivatives in the comparison with the ancestor PAO1 in biofilms. ^*^^*^^*^P < 0.001. (F) Quantification of biofilm biomass formed by PAO1 and two biofilm variants after 24 h of culture in TSB.

To get insights into the genetic bases of evolutionary adaptation to fitness, we sequenced all the evolved populations using an illumina and nanopore-integrated technique. Possibly due to the small scale of evolution, a limited number of mutations were detected (Table 1). Most mutations were mapped in the intergenic regions, and genes encoding ribosomal RNAs or hypothetical proteins with unknown functions. However, two mutations were identified at high frequencies from the populations with improved fitness, including a 3348 nucleotide-long deletion mapped to the *Pseudomonas* filamentous (Pf4) prophage region from B6 with a ratio of 89.1% (Fig. 2A, Table 1), and a non-synonymous SNP converting the 438th amino acid leucine (L) into arginine (R) in BifA from B3 with a frequency of 79.2% (Fig. 2B, Table 1). We herein focused on these two major ones for in-depth analysis, despite the possibility that other mutations with lower frequencies might contribute to fitness improvement. Consistent with the predicted mutation frequency, we determined the proportions of colonies bearing the non-synonymous *bifA* SNP in B3 and the Pf4 large deletion (designated as ΔL) in B6 to be 75.0 and 94.0%, respectively, indicative of strong adaptation to competition fitness (Supplementary Fig. S1C). To better substantiate the adaptative values, we isolated one representative SCV from each of B3 and B6 for experimental characterization. We confirmed the Pf4 large deletion in the B6 variant (designated as B6 variant_ΔL_), and the *bifA* non-synonymous SNP in the B3 variant (designated as B3 variant_*bifA*L438R_) using PCR and Sanger sequencing. Both variants uniformly had a similar growth pattern (Fig. 2C), reduced swarming motility (Fig. 2D), and a higher competition fitness (Fig. 2E), but their biofilm production varied extensively (Fig. 2F). We thus further investigated the physiological bases of fitness improvement caused by these two mutations.

**Table 1:** Mutations detected in all the evolved *P. aeruginosa* populations at the 6th cycle of EE via whole genome sequencing.

**Sample name**	**Locus in PAO1**	**Genomic position**	**Mutation type**	**Mutation**	**Frequency** **(%)**	**Annotation**
P1	Intergenic *PA0148-PA0149*	169 284	InDel	Δ1bp	17.7%	Adenosine deaminase/ECF subfamily sigma-70 factor
P2	*PA0668.1*	723 601	SNP	T → C	31.5%	16S ribosomal RNA
	Intergenic *PA1352-PA1353*	1 467 484	InDel	+G	25.1%	Hypothetical protein/hypothetical protein
P3	Intergenic *tyrZ-PA0668.1*	721 740	SNP	C → T	42.7%	Tyrosine—tRNA ligase/16S ribosomal RNA
	Intergenic *PA1352-PA1353*	1 467 484	InDel	+G	14.4%	Hypothetical protein/hypothetical protein
B1	*PA1458*	1 587 812 to 1 587 977	Deletion	Δ165 bp	6.7%	Two-component sensor
B2	Intergenic *PA0604-PA0605*	667 029	InDel	+C	13.1%	ABC transporter/ABC transporter permease
	Intergenic *tyrZ-PA0668.1*	721 663	InDel	2 bp → CT	17%	Tyrosine—tRNA ligase/16S ribosomal RNA
	*PA0668.1*	723 601	SNP	T → C	36%	16S ribosomal RNA
	*PA0668.4*	726 912	SNP	A → T	21%	23S ribosomal RNA
	Intergenic *PA1352-PA1353*	1 467 484	InDel	+G	9.3%	Hypothetical protein/hypothetical protein
B3	*bifA*	4 895 771	SNP	L438R (CTG → CGG)	79.2%	Protein BifA
	*PA3078*	3 452 123	SNP	Q206H (CAG → CAC)	12.1%	Two-component sensor
	Intergenic *PA1352- PA1353*	1 467 484	InDel	+G	5.4%	Hypothetical protein/hypothetical protein
B4	Intergenic *PA1352-PA1353*	1 467 484	InDel	+G	4.10%	Hypothetical protein/hypothetical protein
	*PA1938*	2 119 350	SNP	W142L (TGG → TTG)	11.9%	Hypothetical protein
B5	Intergenic *PA0604-PA0605*	667 029	InDel	+C	0.7%	ABC transporter/ABC transporter permease
	*PA0668.1*	723 601	SNP	T → C	13%	16S ribosomal RNA
	*PA0668.1*	726 909	SNP	A → T	28%	23S ribosomal RNA
	*PA0668.4*	726 912	SNP	A → T	41%	23S ribosomal RNA
	Intergenic *PA1352-PA1353*	1 467 484	InDel	+G	11%	Hypothetical protein/hypothetical protein
B6	Gene cluster *phrD*-tRNA-gly	785 558 to 788 906	Deletion	Δ 3348 bp	89.1%	Pf4 prophage
	Intergenic *PA0604-PA0605*	667 029	InDel	+C	26%	ABC transporter/ABC transporter permease
	Intergenic *PA1352-PA1353*	1 467 484	InDel	+G	12%	Hypothetical protein/hypothetical protein
	Intergenic *PA1352-PA1353*	1 467 484	InDel	+G	43%	Hypothetical protein/hypothetical protein

Abbreviations: Δ, deletion of nucleotides; +, insertion of nucleotides; →, substitution; SNP, single nucleotide polymorphism.

### Superinfection of *Pseudomonas* filamentous (Pf4) bacteriophage was hijacked to gain a competition advantage in biofilms

**Figure 3:**
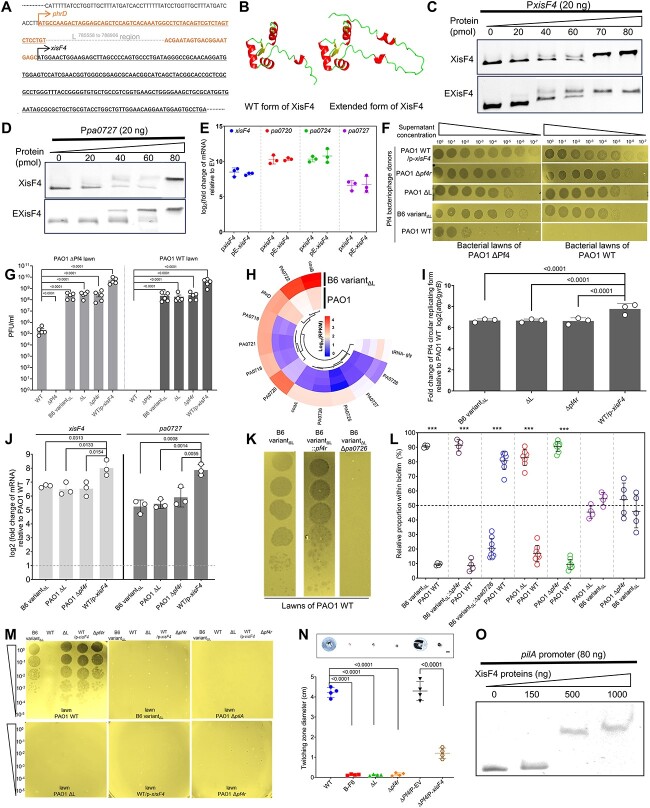
**Superinfection of filamentous bacteriophage Pf4 allowed bacterial adaptation to fitness in biofilms.** (A) Sanger sequencing revealed the region was deleted in the evolved clone B6 variant_ΔL_. The remaining *phrD* and the *xisF4* promoter sequence constitute a 27-amino-acid encoding sequence added upstream of the *xisF4* coding sequence. The underlines indicated the ORFs of *phrD* and *xisF4*, and the arrows indicated the transcriptional starting sites and direction. The dot line represented the deleted region. (B) Structural modeling of the wild type form and the extended form of XisF4 by AlphaFold Protein Structure Database. (C) EMSA showed that the native form and “extended” form of excisionases had a similar affinity to the *xisF4* promoter. (D) EMSA showed that the native form and “extended” form of excisionases had a similar affinity to the *pa0727* promoter. (E) qPCR indicated four Pf4 genes, *xisF4*, *pa0720*, *pa0724*, and *pa0727*, were similarly upregulated by the native form and “extended” form of excisionases relative to empty vector control group tested in the PAO1 Δ*xisF4* strain. (F) Plaques formed by the phage lysates at 12 h post incubation (hpi). (G) Quantification of PFUs on the lawns of PAO1 and ΔPf4. (H) Heatmap indicated most Pf4 prophage genes were differentially expressed between PAO1 and its derivative B6 variant_ΔL_. RPKM, reads per kilobase per million mapped reads. (I) Fold change of Pf4 circular replicating forms in the tested strains relative to PAO1 WT. (J) qPCR indicated *xisF4* and *pa0727* responsible for Pf4 excision and replication were significantly upregulated in the tested strains relative to PAO1 WT. (K) Plaques formed by Pf4 phages released from B6 variant_ΔL_ and its derived mutants on the lawns of PAO1. (L) Pairwise biofilm competition assays with strains co-inoculated in TSB at initially equivalent ratios. ^*^^*^^*^, *P* < 0.001. (M) Plaques formed by the indicated phage lysates on the lawns of various strains at 12 hpi. (N) Twitching motility of various strains on 1.2% agar at 24 hpi. Bar: 1 cm. (O) XisF4 directly bound to the *pilA* promoter. Each experiment was repeated at least three times. Values correspond to means ± standard deviations. Statistical significance was determined using two-tailed unpaired *t*-test or one-way ANOVA with Tukey *post hoc* tests using a 95% confidence interval.

We started by analyzing the Pf4 large deletion, as it reduced biofilm formation but elevated fitness in biofilms. This deletion removed partial *xisF4* promoter sequence and four genes: *phrD, pa0715, pa0716*, and *pf4r* (marked in gray) (Fig. 2A), causing genetic re-organization, and allowing the remaining part of *phrD* and *xisF4* promoter (sequence marked in orange) to constitute a 27-codon sequence added upstream of *xisF4* open reading frame (ORF) (Fig. 3A). This re-organization event generated an “extended” form of XisF4 excisionase (E-XisF4) under the control of the *phrD* promoter (Fig. 3A, B). To assess whether E-XisF4 still retained the DNA-binding activity of the native excisionase, we cloned and purified both proteins for EMSA. The results clearly showed that E-XisF4 had a similar affinity to the well-known targets, the promoters of *xisF4* (Fig. 3C) and *pa0727* (Fig. 3D) [28]. To substantiate this notion, we constructed the PAO1 Δ*xisF4* mutant to exclude the chromosomal *xisF4*, and further induced either of excisionases at an equivalent level in this background. Consequently, transcription of four genes belonging to the XisF4 regulon were found comparably activated, reinforcing that the N-terminal extension had little impact on XisF4 function (Fig. 3E).

The effect of the former three genes on Pf4 was unknown, but Pf4r is a repressor *C* protein that binds to the promoter and represses *xisF4*, a central regulator for Pf4 prophage excision and replication [28, 40]. Loss of *pf4r* would, therefore, induce *xisF4* overexpression and promote excessive production of Pf4 bacteriophages to cause bacterial lysis [28]. This process is called Pf4 superinfection. To verify whether Pf4 superinfection was activated in B6 variant_ΔL_, we quantified active Pf4 particles using the plaque forming unit (PFU) assay. Compared with PAO1, B6 variant_ΔL_ produced ~10^4^ -fold more PFUs on the lawn of PAO1 Pf4 deletion mutant (ΔPf4) (Fig. 3F, G). Pf4 virions from B6 variant_ΔL_ can also form plaques on the PAO1 lawn, but Pf4 from PAO1 itself cannot (Fig. 3F, G). Consistent with the results of the PFU assay, most Pf4 prophage genes were significantly upregulated in B6 variant_ΔL_ (Fig. 3H). The number of Pf4 circular replicative forms also increased (Fig. 3I). These results together indicated that the L deletion removed *pf4r*, de-repressed *xisF4*, and consequently induced Pf4 superinfection. To substantiate this finding, we reconstructed deletions of the L region and *pf4r* separately in PAO1, and found that Pf4 superinfection was indeed activated in both mutants (Fig. 3F, G, I, J). We also monitored the temporal dynamics of *xisF4* expression using the P*xisF4*-*gfp* reporter system, revealing that this gene was upregulated in all the Pf4 super-infective strains with a similar pattern (Supplementary Fig. S2A). Therefore, loss of *pf4r* was likely a major basis for Pf4 superinfection as the phenotypes of Δ*pf4r* closely resembled B6 variant_ΔL_ and also the case of PAO1 with overexpressed *xisF4* (Fig. 3F, G, I, J).

Although Pf4 is a temperate bacteriophage, it would cause bacterial lysis at its super-infective state [26, 28]. We hypothesized that Pf4 superinfection might be hijacked by B6 variant_ΔL_ to repress its competitors in biofilms. To test this, we firstly tried to inhibit *xisF4* in B6 variant_ΔL_ by complementing the cognate repressor Pf4r. However, it had no effect on the Pf4 titer (Fig. 3K) or the competitive fitness (Fig. 3L), likely because the L deletion disrupted the original promoter of *xisF4*, leaving no binding target for Pf4r. Alternatively, deletion of *pa0726* (encoding a membrane protein for Pf4 package and extrusion) [41] blocked Pf4 release of B6 variant_ΔL_ (Fig. 3K) and deprived its competition advantage over PAO1 (Fig. 3L and Supplementary Fig. S2B). Simultaneously, the ΔL and Δ*pf4r* mutants outcompeted PAO1 (Fig. 3L and Supplementary Fig. S2B), but these two mutants grew similarly as B6 variant_ΔL_ in biofilms (Fig. 3L). All these results indicated that Pf4 superinfection was a major physiological basis underpinning the competitive advantage.

We then addressed why B6 variant_ΔL_ and the ΔL and Δ*pf4r* mutants themselves were immune to Pf4 superinfection, as their growth was not repressed (Supplementary Fig. S2C). Given that type IV pili (T4P) is a critical surface receptor of Pf4, we hypothesized that deletions of L or *pf4r* might disrupt T4P expression, thus evading Pf4 recognition. Indeed, deletion of *pilA* encoding the major subunit of T4P in PAO1 (PAO1 Δ*pilA*) enabled resistance to Pf4 superinfection (Fig. 3M). Likewise, B6 variant_ΔL_ and the ΔL and Δ*pf4r* mutants were also immune to Pf4, regardless of its normal or super-infective state (Fig. 3M), suggesting that T4P was inactive in those strains. In support of this indication, the T4P-mediated twitching motility of these Pf4-resistant strains was defective (Fig. 3N). T4P repression in these strains could be ascribed to the loss of transcriptional activation, as both Pf4r [40] and PA0715 [42] in the L region were recently demonstrated to promote transcription of T4P-essential genes. Additionally, Pf4 minor capsid proteins pVII and pIII also interfered with T4P assembly and contributed to Pf4 superinfection exclusion [43, 44]. Furthermore, we examined whether XisF4 had an additional effect on twitching motility, as it was upregulated more than 64-fold in those T4P-defective strains (Fig. 3J). For that, we overexpressed *xisF4* in the PAO1 Δ*Pf4* background and found that the twitching motility significantly declined (Fig. 3N). Considering that XisF4 had a DNA-binding domain [28], it might bind to and repress T4P genes. Indeed, RNA-seq analysis implicated that two essential T4P genes, *pilA* and *pilB*, were significantly downregulated by XisF4 (Supplementary Fig. S2D). Using EMSA, we confirmed the direct binding of XisF4 to the *pilA* promoter (Fig. 3O).

Altogether, the L deletion was the major adaptive mutation conferring the improved competitive fitness to B6 variant_ΔL_. This deletion de-repressed *xisF4* and, in turn, stimulated Pf4 superinfection as an “attacking weapon” to inhibit kin competitors. Simultaneously this deletion inactivated T4P to block Pf4 recognition, which rendered a protective “shield” against Pf4. T4P repression was a synergistic consequence of multiple actions, including the removal of the activators PA0715 and Pf4r, interference of T4P assembly by phage capsid proteins, and transcriptional repression by XisF4.

### A loss-of-function point mutation in *bifA* drove the adaptation to competitive fitness in biofilms

**Figure 4:**
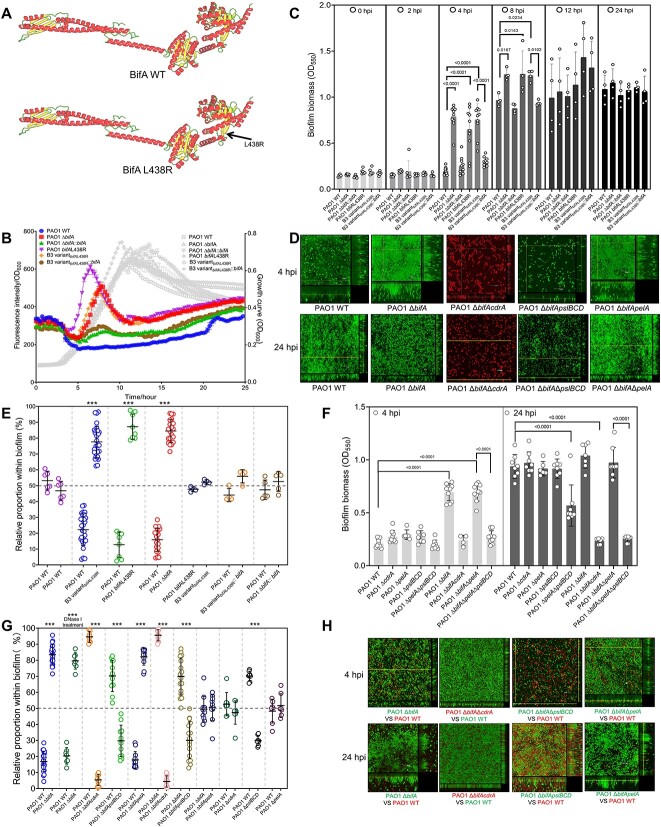
**BifA dysfunction drove bacterial adaptation to competitive fitness via the enhanced expression of CdrA and Psl.** (A) Structural modeling of the wild type BifA and the truncated BifA with L438R predicted using AlphaFold Protein Structure Database. The non-synonymous mutation was marked by the arrow. (B) Intracellular c-di-GMP dynamics of various strains marked in different colors were detected using the P*cdrA-gfp* reporter system (fluorescence intensity units normalized to OD_600_, left Y axis). Bacterial growth detected using OD_600_ (right Y axis) were shown in gray lines. (C) Temporal dynamics of biofilm biomass formed by various strains. (D) Morphology of biofilm grown at 4 and 24 hpi imaged using CLSM. (E) Pairwise biofilm competition assays after 24 h of incubation with strains inoculated at initially equivalent ratios. ^*^^*^^*^, *P* < 0.001, indicated the significant difference in the proportion between the co-cultured strains. (F) Quantification of biofilm biomass at 4 and 24 hpi. (G) Pairwise biofilm competition assays indicated that CdrA and Psl were essential for the competition advantage conferred by BifA deficiency in biofilms at 24 hpi. (H) Biofilm morphology imaged using CLSM. Bacterial fluorophores were indicated by the font colors. Each experiment was repeated at least three times. Values correspond to means ± standard deviations. Statistical significance was determined using two-tailed unpaired *t*-test or one-way ANOVA with Tukey *post hoc* tests using a 95% confidence interval.

We explored the physiological basis for the fitness adaptation of B3 variant_*bifA*L438R_. In *P. aeruginosa*，BifA is a highly conserved PDE responsible for c-di-GMP hydrolysis, so its dysfunction would elevate c-di-GMP and increase biofilm formation [45–47]. Although the point mutation was not predicted to modify BifA structure (Fig. 4A), it was mapped in the EAL domain and thus might interfere with PDE activity (Fig. 2B). To clarify this, we reconstructed the *bifA*L438R point mutation and in-frame deletion separately in PAO1, and then monitored the c-di-GMP dynamics [48]. Like B3 variant_*bifA*L438R_, either of these two mutations elevated c-di-GMP transiently in a narrow growth time window, which strictly corresponded to the exponential (log) phase (Fig. 4B). Meanwhile BifA complementation reduced the c-di-GMP levels comparable to that of PAO1 (Fig. 4B). This result indicated that *bifA*L438R completely abrogated BifA function, and that BifA regulated c-di-GMP and, in turn, biofilm formation in a log phase-responsive manner. Indeed, BifA-negative strains formed more biofilm biomass only at 4- and 8-h post incubation (hpi) (Fig. 4C). Simultaneously, inspection of biofilm morphology by confocal laser scanning microscope (CLSM) also supported that the Δ*bifA* mutant formed a thicker and more structured layer of biofilm at the early attachment stage, but no difference was found at 24 hpi (Fig. 4D).

To validate whether *bifA*L438R was adaptive to competition, we conducted pairwise competition assays. The BifA-negative strains (either Δ*bifA* or *bifA*L438R) were comparably competitive as B3 variant_*bifA*L438R_, but outcompeted PAO1 in biofilms (Fig. 4E). Additionally, BifA complementation into the BifA-negative strains deprived them of the competition advantage over PAO1 (Fig. 4E). Therefore, we concluded that *bifA*L438R was the major adaptive mutation for competition. Moreover, the findings suggested that the placeholder advantage in the mature biofilm was programmed by the transient improvement of early surface adhesion.

BifA dysfunction affected expression of a set of biofilm matrix components (Supplementary Fig. S3A). We thus intended to unravel which specific component was responsible for the competition advantage. The proteinaceous adhesin CdrA is important for PAO1 biofilm [49], and we found that its transcription was upregulated in the BifA mutants (Fig. 4B and Supplementary Fig. S3A). We therefore assessed the impact of CdrA. Consistent with the findings from a previous report [49], *cdrA* deletion in PAO1 WT did not affect biofilm formation (Fig. 4F) or competition (Supplementary Fig. S3B). By contrast, *cdrA* deletion severely impaired biofilm formation of the Δ*bifA* mutant (Fig. 4D, F) and reversed the competitive advantage over PAO1 WT (Fig. 4G, H). CdrA can interact with two distinct EPS, Psl [49], and Pel [50], to promote biofilm formation. Psl is key to facilitating cell-surface adhesion [24, 51, 52], cell–cell communications [53], and bacterial intra−/inter-species competition [54, 55]. We thus investigated the role of Psl in the *bifA* mutant by deleting *pslBCD*. As expected, abrogating Psl production in both WT and Δ*bifA* backgrounds significantly diminished biofilm formation (Fig. 4D, F) and undermined bacterial competitive fitness in biofilm (Fig. 4G, H, and Supplementary Fig. S3B). Comparatively, another EPS Pel appeared dispensable for biofilm formation (Fig. 4D, F) and kin competition (Fig. 4G, H, and Supplementary Fig. S3B) in both the WT and Δ*bifA* backgrounds under the investigated experimental conditions. We further checked the effect of eDNA, as it has important roles in aggregate formation and biofilm development [56, 57]; however, treatment of the WT/Δ*bifA* mixed biofilms with DNase I (to degrade eDNA) did not alter their relative proportion (Fig. 4G). We also reasoned that T4P and flagellum were unlikely to confer the competition advantage as BifA inactivation had little effect on T4P-mediated twitching (Supplementary Fig. S3C) and flagellum-driven swimming motility (Supplementary Fig. S3D). A newly characterized PDE PipA mediated aggregate formation via regulating Pf4 bacteriophage production [36], but we excluded this possibility for the BifA mutants as BifA dysfunction did not alter the PFU quantity (Supplementary Fig. S3E).

To conclude, the mutation *bifA*L438R conferred a competition advantage to B3 variant_*bifA*L438R_ by abolishing BifA protein function, transiently improving c-di-GMP level in the exponential phase, and therefore enhancing expression of two major matrix molecules, the adhesin CdrA and the EPS Psl.

### The commonality in molecular trajectories of fitness adaptation between natural and experimental evolution

**Figure 5:**
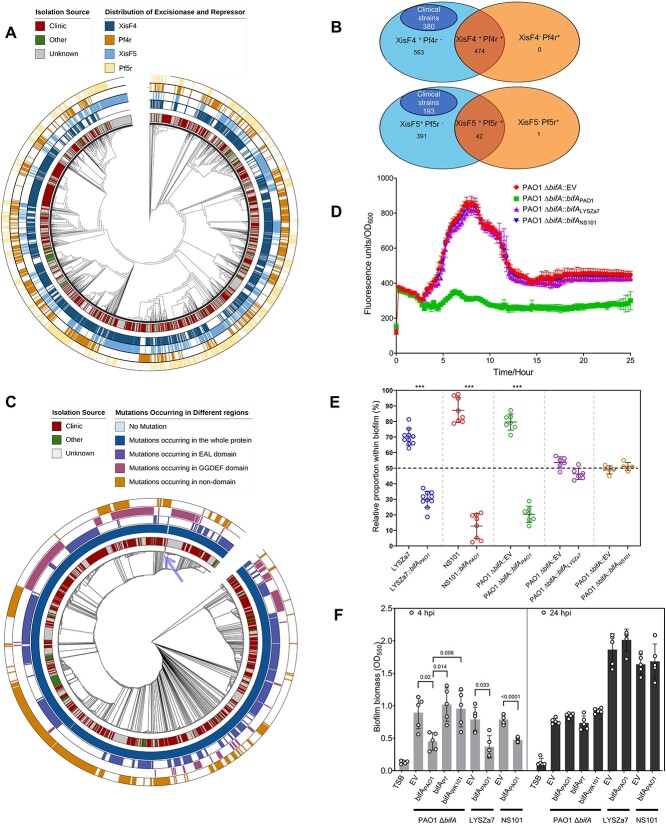
**The molecular strategies driving fitness adaptation in** EE **were prevalent among natural *P. aeruginosa* isolates.** (A) Phylogenetic tree of *coaA* (encoding a Pf4 minor coat protein) homologs. The first to the fifth outer rings from the inside out represent the isolation source, and the presence of *xisF4*, *pf4r*, *xisF5*, and *pf5r*, respectively. (B) Numbers of strains harboring different sets of excisionases XisF4/XisF5 and phage repressors Pf4r/Pf5r. The symbol – shown in the superscript means gene absence and + means gene presence. (C) Phylogenetic tree of BifA homologs. The first to the fifth outer rings from the inside out represent the isolation source, and variations in the whole BifA amino acid sequence, the EAL domain, the GGDEF domain and the non-functional domain, respectively. The clade containing bacterial strains without variation was pointed by the arrow. (D) Intracellular c-di-GMP dynamics of various strains marked in different colors were detected using the P*cdrA-gfp* reporter system (fluorescence intensity units normalized to OD600). (E) Pairwise biofilm competition assays after 24 h of incubation with strains inoculated at initially equivalent ratios. (F) Temporal dynamics of biofilm biomass formed by various strains detected using crystal violet staining. EV, empty control. ^*^^*^^*^, *P* < 0.001, indicated the significant difference in the proportion between the co-cultured strains.

We then investigated whether similar evolutionary strategies could be found in the natural evolution. Loss of the phage repressor *pf4r* was one fundamental adaptive basis to competition, so we analyzed this gene homolog among the Pf prophages from 2645 *P. aeruginosa* strains with their whole genome sequenced. Of them, 1675 were isolated from various clinical specimens, such as sputum, bronchoalveolar lavage fluid (BALF), and blood (Supplementary Table S3). We also included *pf5r* in our analysis because it is a *pf4r* homolog encoded by another Pf subfamily Pf5, displaying a similarly inhibitory effect on the excisionase gene *xisF5* [28]. Alignment of the most similar sequences revealed the prevalence of deletion events in the promoter and ORF of *pf4r* (Supplementary Fig. S4 and Supplementary Table S3) and *pf5r* (Supplementary Fig. S5 and Supplementary Table S3). We thus focused on analyzing the presence and absence of *pf4r* and *pf5r* associated with Pf prophages. To this end, we firstly built a phylogenetic tree based on *coaA* (encoding a minor coat protein of Pf phages) (Fig. 5A). Consistent with the previous phylogenomic analyses [28, 58], the excisionase genes were unevenly distributed among Pf prophages with a prevalence rate of 39.2% (1036/2645) (Fig. 5A and Supplementary Table S3), reinforcing the notion that a large proportion of the Pf4-like phages are likely to be integrated into the host chromosome rather than replicating episomally [28]. Among 1036 Pf prophages with *xisF4*, 563 (54.3%) lost the *pf4r* homologs (Fig. 5B). Similarly, 433 Pf prophages are XisF5-positive, yet 391 of them (90.3%) lost *pf5r* (Fig. 5B and Supplementary Table S3). Taken together, loss of phage repressor genes was a prevalent event observed in more than one half of *P. aeruginosa* strains with Pf prophages integrated into their genomes, and most of them were clinical strains (380 of 563 *xisF4^+^pf4r^−^* strains and 192 of 391 *xisF5^+^pf5r^−^* strains were clinically relevant) (Fig. 5B). This finding suggested that loss of the phage repressors to potentially elicit Pf4/Pf5 superinfection might be a common competition-adaptative strategy, especially for clinical *P. aeruginosa* strains.

To know whether the loss-of-function mutations in BifA prevailed during natural evolution, we searched the PAO1 BifA amino acid sequence against the Pseudomonas Genome Database to obtain homolog sequences from 7482 *P. aeruginosa* strains to construct a phylogenetic tree, which included 4799 clinical isolates (64.1%) (Fig. 5C). Consistent with previous findings, BifA is a widespread and highly conserved PDE, and most strains (6432/7482, 86.0%) had an identical BifA sequence (Supplementary Table S4) [46, 47]. Single amino acid substitution (detected in 1007 strains) was the dominant type of variation, followed by deletion (145 strains) and insertion (13 strains) (Fig. 5C and Supplementary Table S4). We thus investigated whether these variations resulted from gene intrinsic diversity or evolutionary selection by calculating the ratio of non-synonymous substitution rate (dN) to synonymous substitutions rate (dS), a well-established indicator of likely selection pressure [59]. The dN/dS ratio for *bifA* was 2.73, a value greater than 1, suggesting *bifA* is under positive selection during the natural evolution process. Although the BifA GGDEF domain was inactive for c-di-GMP synthesis, it was required together with the EAL domain for the hydrolysis of this molecule [45]. We thus focused on analyzing the variation frequency in those two domains. Given that the loss-of-function *bifA*L438R did not alter the EAL core motif or the known conserved residues [36] (Fig. 2B), we set a stringent criterion that any variation including amino acid substitution, deletion, and insertion in the EAL/GGDEF domains could potentially disrupt enzymatic activity. Based on this assumption, we discovered that 753 strains harbored at least one non-synonymous mutation in the EAL or GGDEF domain, and 427 of them were clinical isolates (Supplementary Table S4 and Fig. 5C). Additionally, we found that isolates carrying such mutations appeared to cluster together in a few major clades rather than distributing evenly in the phylogenetic tree (Fig. 5C), suggesting that the EAL/GGDEF mutations might inactivate BifA protein function and drive diversification during the natural evolution of *P. aeruginosa* isolates. To substantiate this hypothesis, we further characterized *bifA* alleles with putatively loss-of-function mutations from two clinical strains. One strain, LYSZa7, was previously isolated from the sputum samples of a COVID-19 patient, and the other one, NS101, was isolated from BALF samples of an elderly patient with pneumonia [60]. Both clinical strains harbored non-synonymous mutations in the EAL domain, with *bifA*A479E for LYSZa7 and *bifA*L440Q for NS101 (Fig. 2B). Individual introduction of these *bifA* alleles into PAO1 Δ*bifA* failed to cause any significant change in c-di-GMP levels (Fig. 5D), or competition fitness (Fig. 5E), or biofilm formation (Fig. 5F), indicating that the mutations are detrimental for BifA function. Moreover, complementation of the active BifA (from PAO1) into the clinical strains reduced the early biofilm attachment (Supplementary Fig. S3F) and undermined the fitness in biofilms (Fig. 5E). Therefore, these experimental data supported the hypothesis that mutations changing the conserved sequence in EAL or GGDEF domain could disrupt protein function and thus drive evolutionary adaptation to competitive fitness.

### The competitive fitness-adaptive mutations reduced bacterial virulence

**Figure 6:**
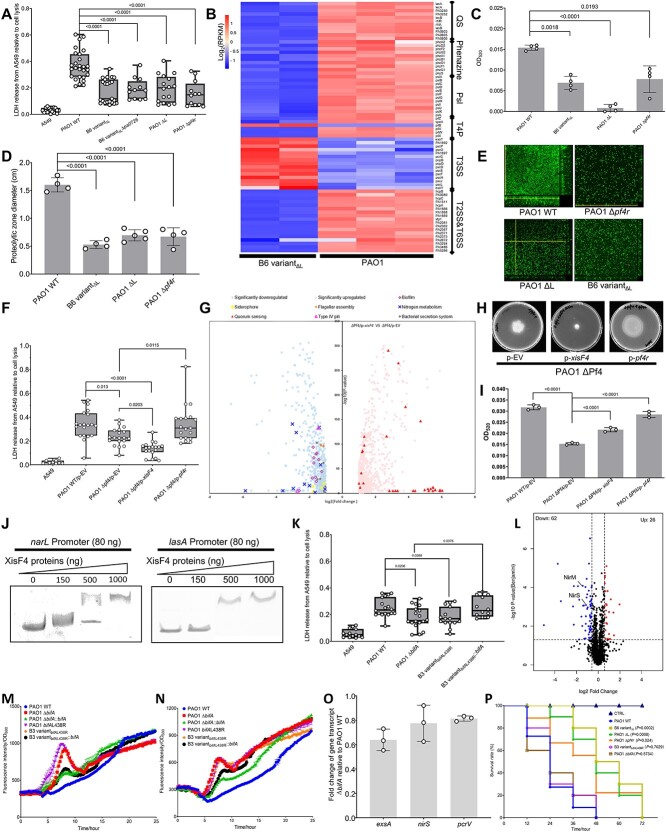
**The kin competition-adaptive mutations reduced the virulence of *P. aeruginosa*.** (A) Box plot diagram showing LDH activity of A549 cells after 6 h infection with different strains at multiplicity of infection (MOI) = 10:1, values were shown as a percentage compared with a lysis control. (B) Heatmap showing the virulence genes differentially expressed in B6 variant_ΔL_ relative to PAO1. RPKM, reads per Kilobase per million mapped reads. (C) Quantification of pyocyanin from various *P. aeruginosa* strains after 24 h culture. (D) Extracellular protease production of various *P. aeruginosa* strains after 8 h incubation. (E) Morphology of biofilm at 24hpi imaged using CLSM. (F) Box plot diagram showing LDH activity of A549 cells after 6 h infection with different strains transformed with pHERD20^T^ expression system at MOI = 10:1, values were expressed as a percentage compared with a lysis control. (G) Volcano plot showed the genes differentially expressed between ΔPf4/p-EV and ΔPf4/p-*xisF4*. (H) Swimming motility mediated by flagellum was quantified on 0.6% agar plates after 24 h of incubation. (I) Quantification of pyocyanin production from various *P. aeruginosa* strains transformed with pHERD20^T^ expression system. (J) EMSA showed that XisF4 can bind to the promoters of *narL* and *lasA*. (K) Box plot diagram showing the LDH activity of A549 cells following 6 h incubation with different *P. aeruginosa* strains (MOI = 10:1). (L) Volcano plot of the differentially expressed proteins between PAO1 and B3 variant_*bifA*L438R_. Dynamics of (M) *rsmY* and (N) *rsmZ* expression indicated by the reporter system P*rsmY-gfp* and P*rsmZ-gfp,* respectively. (O) Relative transcript levels of *nirS, exsA*, and *pcrV* in the Δ*bifA* mutant normalized against the PAO1 WT. (P) Survival rates of *G. mellonella* larvae (*n* = 30 per group) infected by various *P. aeruginosa* strains. CTRL indicates the control group of larvae without *P. aeruginosa* injection. The comparison of survival curves between the PAO1 WT-infected group and other mutants or evolved clones was performed using Log-rank (Mantel–Cox) test. *P* values were indicated after the strains. Each experiment was conducted at least three times. EV, empty control. Values correspond to means ± standard deviations. Statistical significance in other experiments was determined using one-way ANOVA with Tukey *post hoc* tests using a 95% confidence interval.

To scrutinize the impact of these fitness-adaptive mutations on the bacterial pathogenicity, we challenged human alveolar epithelial cells A549 with various bacterial strains and measured cell death by determining the cytosolic release of lactate dehydrogenase (LDH). Compared with PAO1, the ΔL and Δ*pf4r* mutants, and the evolved clone B6 variant_ΔL_ were significantly less cytotoxic (Fig. 6A). Through transcriptomic analysis, we found many genes enriched in virulence, such as QS, the bacterial secretion systems, and chemotaxis, were downregulated in B6 variant_ΔL_ (Fig. 6B). Consistent with the transcriptomic data, the production of two QS-controlled metabolites, pyocyanin (Fig. 6C) and extracellular protease (Fig. 6D), was significantly reduced in B6 variant_ΔL_ as well as in the ΔL and Δ*pf4r* mutants. Although it was unclear whether *pa0716* is related to virulence, the other genes in the L region (*phrD*, *pa0715*, and *pf4r*) encoded a small RNA and two proteinaceous regulators that are all known to promote QS and motility [40, 42, 61]. The losses of these regulators, therefore, impaired bacterial cytotoxicity and biofilm formation. This finding also explained why B6 variant_ΔL_ and the ΔL or Δ*pf4r* mutants developed less biofilm compared with PAO1 (Fig. 6E). Moreover, *pa0726* deletion in B6 variant_ΔL_ had minimal effect on cytotoxicity (Fig. 6A), suggesting that at least in the mammalian cell infection model, deletion of the L region or *pf4r* impaired bacterial virulence largely due to the suppression of virulence factors rather than the absence of bacteriophage particles. Furthermore, we evaluated the effect of *xisF4* on global virulence as it has been implicated to regulate T4P as demonstrated above. *pf4r* overexpression in PAO1 ΔPf4, here used as a positive control, elevated LDH release, in contrast *xisF4* overexpression induced a lower level of LDH, indicative of a repressive effect of *xisF4* on virulence (Fig. 6F). RNA-seq analysis revealed that *xisF4* downregulated virulent genes associated with the protein secretion systems, biofilm, siderophore, T4P, nitrate metabolism, and flagellum assembly, but upregulated QS (Fig. 6G). RT-qPCR validated the transcriptomic data by determining the expression changes of four representative virulence genes (Supplementary Fig. S6). It also reinforced the finding that E-XisF4 had a similar regulatory efficacy to the native form of XisF4. Consistently, the flagellum-mediated swimming motility was repressed by XisF4 (Fig. 6H), whereas the QS-controlled pyocyanin production was elevated (Fig. 6I). EMSA further showed that purified XisF4 can bind directly to the promoters of *narL* and *lasA* (Fig. 6J), the representative genes in nitrate metabolism and QS pathways, respectively. Therefore, the excisionase XisF4 was a global regulator directly repressing multiple virulence pathways but increasing QS.

Likewise, the Δ*bifA* mutant and B3 variant_*bifA*L438R_ were less virulent than PAO1 WT, whereas *bifA* complementation restored the cytotoxicity to the PAO1 level (Fig. 6K). These results indicated that BifA contributed to bacterial virulence, despite a transient c-di-GMP modulation. To gain insights in which virulence factors were affected by the BifA-controlled c-di-GMP system, we performed a proteomics analysis. Although a limited number of proteins were deferentially expressed, NirS and NirM that are necessary for nitrogen metabolism were significantly downregulated in the Δ*bifA* mutant relative to the WT (Fig. 6L and Supplementary Table S5). We obtained similar results when comparing B3 variant_*bifA*L438R_ to WT (Supplementary Fig. S7 and Supplementary Table S6). In *P. aeruginosa*, the nitrate reductase NirS is required for fully activating type III secretion system (T3SS) [62]. Moreover, using the reporter systems, we found that two important regulatory RNAs, *rsmZ* (Fig. 6M), and *rsmY* (Fig. 6N), were induced transiently due to BifA dysfunction. These two RNAs are important for sequestering the virulence enhancer protein RsmA from its target mRNAs, thus repressing T3SS but promoting biofilm formation [63–65]. These findings together implied that the transiently induced c-di-GMP pool in the Δ*bifA* mutant modulated expression of *rsmZ/Y* and nitrate reductase NirS, and, in turn, downregulated T3SS, the critical virulent factor for acute *P. aeruginosa* infections [66]. Indeed, expression of T3SS-associated genes, *exsA, nirS*, and *pcrV*, were slightly downregulated in the Δ*bifA* mutant (Fig. 6O).

To finally confirm the effect of the fitness-adaptive mutations on bacterial virulence *in vivo*, we monitored survival of *G. mellonella* larvae over time following bacterial infection. The control group devoid of bacterial injection showed 100% viability, yet PAO1 infection caused rapid death within 48 hpi (Fig. 6P). BifA dysfunction had no impact on bacterial virulence, possibly due to the transient action of BifA on c-di-GMP. By contrast, infection by *P. aeruginosa* strains with Pf4 superinfection resulted in enhanced survival within 72 h, indicating a lower level of pathogenesis. Overall, the fitness-adaptive mutations compromised bacterial virulence but to different extents.

## Discussion

Biofilm is a group of highly dense bacterial cells that are distributed in a heterogenous 3D space with limited access to nutrient and oxygen [3–6]. These properties per se allowed biofilm to be an incubator of genotypic and phenotypic diversity [11–14], which further drive evolutionary adaptation. In alignment with prior analogous studies, this work reveals that divergent molecular strategies can drive evolutionary adaptation of *P. aeruginosa* biofilms to competitive fitness. Adhesion is one of the most extensively-studied weapon in microbial competition [67]. Production of adhesive molecules is tightly regulated by c-di-GMP, which is, in turn, determined by a sophisticated network of DGCs and PDEs in response to environmental cues [22, 23]. Therefore, mutations altering c-di-GMP dynamics as shown in this work and previous studies [13, 16, 17, 68, 69], or directly modulating production of the downstream EPS [16, 55] and motility [14, 69] can enhance biofilm attachment and thus confer a placeholder advantage. However, evolved biofilm variants with either higher or lower c-di-GMP levels were both found in the past studies [13, 60], suggesting the possibility of alternative strategies for niche occupation. Recent evolution experiments revealed the involvement of *P. aeruginosa* bacteriophage [16, 69]. Indeed, many mutations causing alterations of phage production [16] or resistance [16, 69, 70] have been detected in the *P. aeruginosa* biofilm variants, but how they specifically contributed to biofilm evolution remained unknown. In this work we dissected the contributing roles of Pf4 bacteriophage superinfection in fitness adaptation. Pf4 bacteriophages can diversify at an evolutionary rate comparable to RNA viruses, far faster than the rest of bacterial genome [16, 40]. This explains why Pf4 mutations emerge so rapidly during the evolution of *P. aeruginosa* biofilms. Apart from the abovementioned strategies, a distinct approach associated with competition adaptation is through pyocin*.* Pyocins are, by nature, bacteriocins produced by *P. aeruginosa* strains to kill their susceptible kin competitors [71]. Three major types of pyocins have been identified: S-, R-, and F-pyocins. The S-pyocins are large multidomain polypeptides that can bind to cognate immunity proteins to inactivate the catalytic domain, whereas resistance to R- and F- pyocins is mediated through incompatible lipopolysaccharide. Therefore, lipopolysaccharide structural alteration allowing resistance to R-pyocins [72], or large genomic deletions causing pyomelanin synthesis and concomitant resistance to S-pyocins [73], can drive adaptation to competitive fitness. Furthermore, a different but also important tactic is cheating, by which the beneficial substances secreted by producers can be scavenged by exploitative non-producers (also known as cheaters) without paying metabolic costs [74–77]. The importance of this strategy is nicely exemplified by the high prevalence of loss-of-function mutations in QS regulators. For instance, *lasR* mutants were frequently detected in biofilm variants that evolved *in vitro* or *in vivo*. [77–80]. The divergence in the molecular strategies for fitness adaptation in biofilms across studies vividly illustrates the bacterial genome plasticity.

In this work, we demonstrated the lifestyle conversion of Pf4 bacteriophage from lysogeny to lysis was reconfigured and exploited by the bacterial host to gain fitness adaptation in biofilms. The genetic deletion in the Pf4 region removed four genes including the repressor *C* protein Pf4r, resulting in the de-repression of the core regulator *xisF4* to stimulate the Pf4 superinfection and a concomitant resistance to Pf4 reinfection. How Pf superinfection causes bacterial lysis is still unclear, but it must rely on a functional T4P to initiate infection, and a large number of virions to circumvent the phage immunity conferred by the repressor proteins (like Pf4r) [28, 43]. Although it is rare for the ssDNA virus to carry its own excisionase, the Pf-encoded excisionase XisF4 and its homolog XisF5 are widespread among Pf phages in *P. aeruginosa* [28]. Excision and release of Pf prophages (like Pf4 in PAO1) are supposed to be finely tuned and well controlled, as they are otherwise metabolically costly and would impair bacterial fitness. Within the harsh conditions of a biofilm, however Pf superinfection turns out to be a potent strategy for bacterial hosts to outcompete their kins for nutrients and space. Our work uncovered that the loss of the cognate repressor to de-repress the excisionase and stimulate Pf superinfection was a prevalent evolutionary event among clinical *P. aeruginosa* strains.

In this work, we also found that the c-di-GMP-related gene *bifA* is under positive selection during *P. aeruginosa* biofilm evolution. In *P. aeruginosa* PAO1, there are 17 different proteins with a DGC domain, 8 with a PDE domain, and 16 with both domains [81]. Intriguingly, impacts of the dual-domain proteins on c-di-GMP and biofilm formation are heterogeneous, or even opposing [81–83]. BifA is one such dual-domain protein [47], and its PDE activity requires both GGDEF and EAL domains [45]. In this work, we found that the *bifA*L438R mutation abolished PDE activity and elevated the c-di-GMP levels transiently in the log growth phase. It was just such a small signal modulation that significantly promoted early bacterial adhesion and programmed a drastic placeholder advantage in biofilms. This molecular trajectory seemed highly prevalent in natural evolution, as an extraordinarily high level of variation was detected in the BifA GGDEF and EAL domains in a vast number of clinical *P. aeruginosa* isolates. Furthermore, we addressed another interesting issue that the *bifA* defect increased biofilm formation and rendered a competition advantage mainly through enhanced production of the EPS Psl and the adhesin CdrA. CdrA is dispensable for static biofilm formation in PAO1 and some isogenic PDE-defective mutants (Δ*wspF* and Δ*pipA*) [36, 49], possibly due to the masking effect of EPS [84]. Nevertheless, CdrA and Psl were both important for biofilm development and competitive fitness on a BifA-defective background. Both CdrA and Psl have cell-associated forms and cell-free forms [49, 85], but we consider that the cell-associated forms play major roles as they cannot be exploited by competitors.

Another important finding from our work is elucidating the inhibitory effect of the competition-adaptive mutations on bacterial virulence. For B6 variant_ΔL_, the L deletion significantly reduced bacterial virulence, as most genes in this region (*phrD, pa0715*, and *pf4r*) encode virulence enhancers [40, 42, 61]. Adding to the complexity, our findings demonstrated that the phage excisionase XisF4 serves not only as a transcriptional activator of Pf4 prophage genes, but also a master regulator of virulence genes. Overall, XisF4 reduced bacterial pathogenesis, although it upregulated QS-associated genes. These findings extend our understanding of the prophage genes clustered at the 5′-proximal side of Pf4 prophage. Most of these genes have important and pleotropic effects on multiple physiological traits of the bacterial host. Pioneering works proposed that bacterial virulence was reduced in the ΔPf4 mutant due to the absence of Pf4 virions [26, 86], but here we showed that the loss of these regulatory proteins also contributed to the virulence reduction. For the other adaptive derivative B3 variant_*bifA*L438R_, the molecular mechanism for virulence reduction is simpler. Generally, deletion of *dgc* genes may induce a lower level of c-di-GMP to favor an acute infection via T3SS; however deletion of *pde* genes results in high c-di-GMP levels to shift to a chronic infection [87]. Here, the finding that BifA inactivation transiently elevated c-di-GMP and, in turn, slightly downregulated T3SS could explain why the *bifA* mutants were less cytotoxic in the acute cell infection model, whereas it was similarly virulent to PAO1 WT in the chronic infection model based on *G. mellonella*. However, this principle does not fit all the *dgc/pde* genes, especially for *P. aeruginosa* harboring a complex of DGC/PDE.

Parallelism in the adaptation strategies across lineages is lacking within this study, possibly due to a limited number of evolution cycles. However, it is strongly implicated between our experiment and the prior ones [16, 17, 30, 88], and also between the *in vitro* evolution and the real-world evolution of clinical strains. This parallelism substantiates that the biofilm environment is intrinsically a powerful driving force for *P. aeruginosa* evolution.

## Supplementary Material

Supplementary_Table_1_wrae135

Supplementary_Table_2_wrae135

Supplementary_Table_3_wrae135

Supplementary_Table_4_wrae135

Supplementary_Table_5_wrae135

Supplementary_Table_6_wrae135

Supplementary_materials_wrae135

## Data Availability

Whole genome re-sequencing data were submitted to Genbank under the Bioproject ID: PRJNA1050259, PRJNA1104558, and PRJNA1101207. The RNA-seq data of *P. aeruginosa* that support the findings of this study have been deposited in the NCBI Sequence Read Archive (SRA) under accession numbers PRJNA1049987. Additional data that support the findings of this study are provided in the supplementary files.
