# Supplementary material for: Divergent molecular strategies drive evolutionary adaptation to competitive fitness in biofilm formation
Source: ISME J. 2024 Jul 25;18(1):wrae135. doi: 10.1093/ismejo/wrae135 (PMC11307329; doi:10.1093/ismejo/wrae135)
Supplement: Supplementary_Table_1_wrae135 [file supplementary_table_1_wrae135.docx]

**Supplementary Table 1. Strains and plasmids used in this study**

| **Strain or plasmid** | **Relevant genotype and/or characteristics** | **Reference** |
| --- | --- | --- |
| ***Escherichia coli strains*** |  |  |
| *E. coli* TOP10 | F-, *mcrA* Δ(*mrr*-*hsd* RMS-*mcr* *BC*) *φ80lacZ* Δ*M15* Δ*lac* *X74* *recA1* *ara* Δ*139* Δ(*ara*-l*eu*) *7697* *galU* *galK* *rpsL* (Strr) *endA1* *nupG* | TIANGEN |
| *E. coli* RK600 | Cm^R^, ColE1, oriV, RK2, mob^+^, tra^+^; helper plasmid in triparental mating | [[1](#_ENREF_1)] |
| *E. coli* BL21 (DE3) | F– *omp*T *hsdS_B_* (rB–, mB–) *gal dcm* (DE3) | Novagen |
| ***Pseudomonas aeruginosa* strains** |  |  |
| PAO1 | *Pseudomonas aeruginosa* ATCC15692 | [[2](#_ENREF_2)] |
| P1 | The 6^th^ cycle evolved population 1 from planktonic culture of PAO1 | This study |
| P2 | The 6^th^ cycle evolved population 2 from planktonic culture of PAO1 | This study |
| P3 | The 6^th^ cycle evolved population 3 from planktonic culture of PAO1 | This study |
| B1 | The 6^th^ cycle evolved population 1 from biofilm culture of PAO1 | This study |
| B2 | The 6^th^ cycle evolved population 2 from biofilm culture of PAO1 | This study |
| B3 | The 6^th^ cycle evolved population 3 from biofilm culture of PAO1 | This study |
| B4 | The 6^th^ cycle evolved population 4 from biofilm culture of PAO1 | This study |
| B5 | The 6^th^ cycle evolved population 5 from biofilm culture of PAO1 | This study |
| B6 | The 6^th^ cycle evolved population 6 from biofilm culture of PAO1 | This study |
| B3 variant *_bifA_*_L438R_ | A derivate from the 6^th^ cycle evolved population 3 from biofilm culture of PAO1 | This study |
| B6 variant_ΔL_ | A derivative from the 6^th^ cycle evolved population 6 from biofilm culture of PAO1 | This study |
| PAO1 Δ*L* | Deletion of a genetic region from the genomic position 785558 to 788906 in PAO1 | This study |
| PAO1 Δ*pf4r* | In-frame deletion of *pf4r* in PAO1 | This study |
| PAO1 Δ*xisF4* | In-frame deletion of *xisF4* in PAO1 | This study |
| B6 variant_ΔL_:: *pf4r* | Complementation of *pf4r* into evolved strain B6 variant_ΔL_ | This study |
| B6 variant_ΔL_ Δ*pa0726* | In-frame deletion of *pa0726* in evolved strain B6 variant_ΔL_ | This study |
| PAO1 Δ*pilA* | In-frame deletion of *pilA* in PAO1 | This study |
| PAO1 *bifA*L438R | PAO1 with a non-synonymous SNP in *bifA* | This study |
| PAO1 Δ*bifA* | In-frame deletion of *bifA* in PAO1 | This study |
| PAO1 Δ*bifA::bifA* | Complementation of *bifA* into PAO1 Δ*bifA* | This study |
| PAO1 Δ*bifA::bifA* _LYSZa7_ | Complementation of the *bifA* allele from the clinical strain LYSZa7 into PAO1 Δ*bifA* | This study |
| PAO1 Δ*bifA::bifA* _NS101_ | Complementation of the *bifA* allele from the clinical strain NS101 into PAO1 Δ*bifA* | This study |
| B3 variant *_bifA_*_L438R_::*bifA* | Complementation of *bifA* into evolved strain B3 variant *_bifA_*_L438G_ | This study |
| PAO1 Δ*cdrA* | In-frame deletion of *cdrA* in PAO1 | This study |
| PAO1 Δ*pslBCD* | In-frame deletion of *pslBCD* in PAO1 | [[3](#_ENREF_3)] |
| PAO1 Δ*pelA* | In-frame deletion of *pelA* in PAO1 | [[3](#_ENREF_3)] |
| PAO1 Δ*bifA*Δ*cdrA* | *bifA* and *cdrA* double-mutant derived from PAO1 | This study |
| PAO1 Δ*bifA*Δ*pslBCD* | *bifA* and *pslBCD* double-mutant derived from PAO1 | This study |
| PAO1 Δ*bifA*Δ*pelA* | *bifA* and *pelA* double-mutant derived from PAO1 | This study |
| LYSZa7 | A clinical *P. aeruginosa* strain isolated from sputum samples of a COVID-19 patient | This study |
| NS101 | A clinical *P. aeruginosa* strain isolated from BALF samples of an elder patient with pneumonia | This study |
| **Plasmids** |  |  |
| pK18 | Small mobilizable vector, Gm^R^, sucrose sensitive (*sacB*) | [[4](#_ENREF_4)] |
| pBF13 | R6K replicon-based helper plasmid, providing the Tn7 transposition function in *trans*; Amp^R^, mob+ | [[5](#_ENREF_5)] |
| pUCP22::*cdrA-gfp* | *cdrA* promoter fused to *gfp*; Carb^R^ | [[6](#_ENREF_6)] |
| mini-CTX1 | Genetic complementation plasmid; Tc^R^ | [[7](#_ENREF_7)] |
| mini-CTX1::*pf4r* | *pf4r* in mini-CTX1 HindIII/BamHI) ; Tc^R^ | This study |
| mini-CTX1::*bifA* | *bifA* in mini-CTX1 HindIII/BamHI) ; Tc^R^ | This study |
| mini Tn7-*gfp* | Gm^R^ on mini-Tn7^T^; mobilizable; for *gfp* tagging | [[8](#_ENREF_8)] |
| mini Tn7-mCherry | Gm^R^ on mini-Tn7^T^; mobilizable; for mCherry tagging | [[9](#_ENREF_9)] |
| pFLP2 | Plasmid harboring the inducible flp recombinase; Ap^R^ (Cb^R^). | [[8](#_ENREF_8)] |
| pHERD20^T^ | Expression vector with *araC*-P_BAD_ promoter; Carb^R^ | [[10](#_ENREF_10)] |
| pHERD20^T^/p-*xisF4* | *xisF4* in pHERD20^T^ EcoRI/HindIII, Ap^R^ | This study |
| pHERD20^T^/p-E*xisF4* | *Extended form of xisF4* in pHERD20^T^ EcoRI/HindIII, Ap^R^ | This study |
| pHERD20^T^/p-*pf4r* | *pf4r* in pHERD20^T^ EcoRI/HindIII, Ap^R^ | This study |
| pET28a | Expression vector, Km^R^ | Novagen |
| pET28a-*xisF4* | *xisF4* in pET28a XhoI/EcoRI, Km^R^ | This study |
| pET28a-*xisF4* (extended) | Extended form of *xisF4* in pET28a XhoI/EcoRI, Km^R^ | This study |
| pUCP22::*rsmY-gfp* | *rsmY* promoter fused to *gfp*; Carb^R^ | [[11](#_ENREF_11)] |
| pUCP22::*rsmZ-gfp* | *rsmZ* promoter fused to *gfp*; Carb^R^ | [[11](#_ENREF_11)] |

**References**

1. Kessler B, de Lorenzo V,Timmis KN. A general system to integratelacZ fusions into the chromosomes of gram-negative eubacteria: regulation of the Pm promoter of theTOL plasmid studied with all controlling elements in monocopy*.* Mol Genet Genom 1992; **233**: 293-301. https://doi.org/10.1007/BF00587591

2. Hentzer M, Riedel K, Rasmussen TB *et al*. Inhibition of quorum sensing in *Pseudomonas aeruginosa* biofilm bacteria by a halogenated furanone compound*.* Microbiology. 2002; **148**: 87-102. https://doi.org/10.1002/jobm.201500268

3. Cai YM, Yu KW, Liu JH *et al*. The c-di-GMP phosphodiesterase PipA (PA0285) regulates autoaggregation and Pf4 bacteriophage production in *Pseudomonas aeruginosa* PAO1*.* Appl Environ Microbiol. 2022; **88**: e0003922. https://doi.org/10.1128/aem.00039-22

4. Schäfer A, Tauch A, Jäger W *et al*. Small mobilizable multi-purpose cloning vectors derived from the *Escherichia coli* plasmids pK18 and pK19: selection of defined deletions in the chromosome of *Corynebacterium glutamicum.* Gene. 1994; **145**: 69-73. https://doi.org/10.1016/0378-1119(94)90324-7

5. Bao Y, Lies DP, Fu H *et al*. An improved Tn7-based system for the single-copy insertion of cloned genes into chromosomes of gram-negative bacteria*.* Gene. 1991; **109**: 167-8. https://doi.org/10.1016/0378-1119(91)90604-a

6. Rybtke MT, Borlee BR, Murakami K *et al*. Fluorescence-based reporter for gauging cyclic di-GMP levels in *Pseudomonas aeruginosa.* Appl Environ Microbiol. 2012; **78**: 5060-9. https://doi.org/10.1128/AEM.00414-12

7. Hoang TT, Kutchma AJ, Becher A *et al*. Integration-proficient plasmids for *Pseudomonas aeruginosa*: Site-specific integration and use for engineering of reporter and expression strains*.* Plasmid. 2000; **43**: 59-72. https://doi.org/10.1006/plas.1999.1441

8. Choi K-H, Gaynor JB, White KG *et al*. A Tn7-based broad-range bacterial cloning and expression system*.* Nat Methods. 2005; **2**: 443-8. https://doi.org/10.1038/nmeth765

9. Lagendijk EL, Validov S, Lamers GEM *et al*. Genetic tools for tagging Gram-negative bacteria with mCherry for visualization in vitro and in natural habitats, biofilm and pathogenicity studies*.* FEMS Microbiol Lett. 2010; **305**: 81-90. https://doi.org/10.1111/j.1574-6968.2010.01916.x

10. Qiu D, Damron FH, Mima T *et al*. P_BAD_-based shuttle vectors for functional analysis of toxic and highly regulated genes in *Pseudomonas* and *Burkholderia* spp. and other bacteria*.* Appl Environ Microbiol. 2008; **74**: 7422-6. https://doi.org/10.1128/AEM.01369-08

11. Chua SL, Liu Y, Yam JKH *et al*. Dispersed cells represent a distinct stage in the transition from bacterial biofilm to planktonic lifestyles*.* Nat Commun. 2014; **5**: 4462. https://doi.org/10.1038/ncomms5462
