# Supplementary material for: Divergent molecular strategies drive evolutionary adaptation to competitive fitness in biofilm formation
Source: ISME J. 2024 Jul 25;18(1):wrae135. doi: 10.1093/ismejo/wrae135 (PMC11307329; doi:10.1093/ismejo/wrae135)
Supplement: Supplementary_materials_wrae135 [file supplementary_materials_wrae135.pdf]

Supplementary materials

Divergent molecular strategies drive evolutionary adaptation to competitive fitness in biofilm formation

Mingxing Tang[1*#], Ruixue Yang[2*], Zilin Zhuang[3*], Shuhong Han[3], Yunke Sun[3], Peiyu Li[4], Kewei Fan[4], Zhao Cai[5], Qiong Yang[1], Zhijian Yu[4#], Liang Yang[3#], Shuo Li[1,6#]

1. Department of Otorhinolaryngology, Nanshan People's Hospital, Shenzhen, China
2. Community Health Service Center of Southern University of Science and Technology, Nanshan Medical Group, Shenzhen, China
3. Department of Pharmacology, School of Medicine, Southern University of Science and Technology, Shenzhen, China
4. Department of Infectious Diseases, Nanshan People's Hospital, Shenzhen University School of Medicine, China
5. Shenzhen Mindray Bio-Medical Electronics Co., Ltd
6. Allergy Prevention and Control Center, Nanshan People's Hospital, Shenzhen, China

#Correspondence to Shuo Li (shuoli@email.szu.edu.cn); Liang Yang (yangl@sustech.edu.cn); ZhiJian Yu (yuzhijiansmu@163.com); Mingxing Tang (tangstar2013@163.com)

* Mingxing Tang, Ruixue Yang, and Zilin Zhuang contributed equally to this work.

Running title: Divergent strategies for fitness adaptation

Keywords: Biofilm evolution; Bacteriophage superinfection; c-di-GMP signaling; Bacterial virulence; Bioinformatic analysis

Contents

Supplementary Methods

Pf4 replicative-form (RF) quantification

RT-qPCR and RNA-seq

Proteomic analysis

Protease assay

Bioinformatics analysis

Biofilm biomass and morphology observation using Confocal Laser Scanning Microscope

Plaque assay

Motility assays

Alpha2 model of protein structure

Protein expression and purification

Electrophoretic mobility shift assay (EMSA)

Transcriptional reporter assays

Pyocyanin measurement

Cell culture and cytotoxicity assay

In vivo infection of *Galleria mellonella* larvae

Supplementary Figures

Supplementary Fig. 1: Two fitness-adaptative mutations emerged in the experimental evolution of *Pseudomonas aeruginosa* PAO1 biofilm.

Supplementary Fig. 2: A large deletion in the Pf4 prophage region caused Pf4 superinfection and a concomitant resistance to Pf4, thus conferring the fitness adaptation to the evolved clone B6 variant$_{\Delta L}$ in biofilms.

Supplementary Fig. 3: A non-synonymous mutation in *bifA* conferred a biofilm competition advantage to a *P. aeruginosa* PAO1 evolved derivative, whereas the Pel exopolysaccharide, type IV pili, flagellum and Pf4 phages were not involved.

Supplementary Fig. 4: Gene sequence alignment of *pf4r*.

Supplementary Fig. 5: Gene sequence alignment of *pf5r*.

Supplementary Fig. 6: Expression fold changes of four representative virulence genes detected by RT-qPCR in PAO1 Δ*xisF4* with either *xisF4* or E-*xisF4* induced with 2 M arabinose under the control of an inducible promoter P_{BAD}.

Supplementary Fig. 7: Volcano plot showing the differentially expressed proteins in PAO1 Δ*bifA*

relative to the PAO1 wild type strain.

Supplementary Tables:

Table S1: Strains and plasmids used in this study.

Table S2: Primers used in this study.

Table S3: *Pseudomonas aeruginosa* strains used for the *coaA*-based phylogeny and the presence/absence analysis of genes encoding excisionases and phage repressors.

Table S4: *Pseudomonas aeruginosa* strains used for the BifA-based phylogeny and variation type analysis.

Table S5: Differentially expressed proteins in PAO1 Δ*bifA* relative to the PAO1 wild type strain.

Table S6: Differentially expressed proteins in B3 variant$_{\mathit{bifA}\mathrm{L438R}}$ relative to the PAO1 wild type strain.

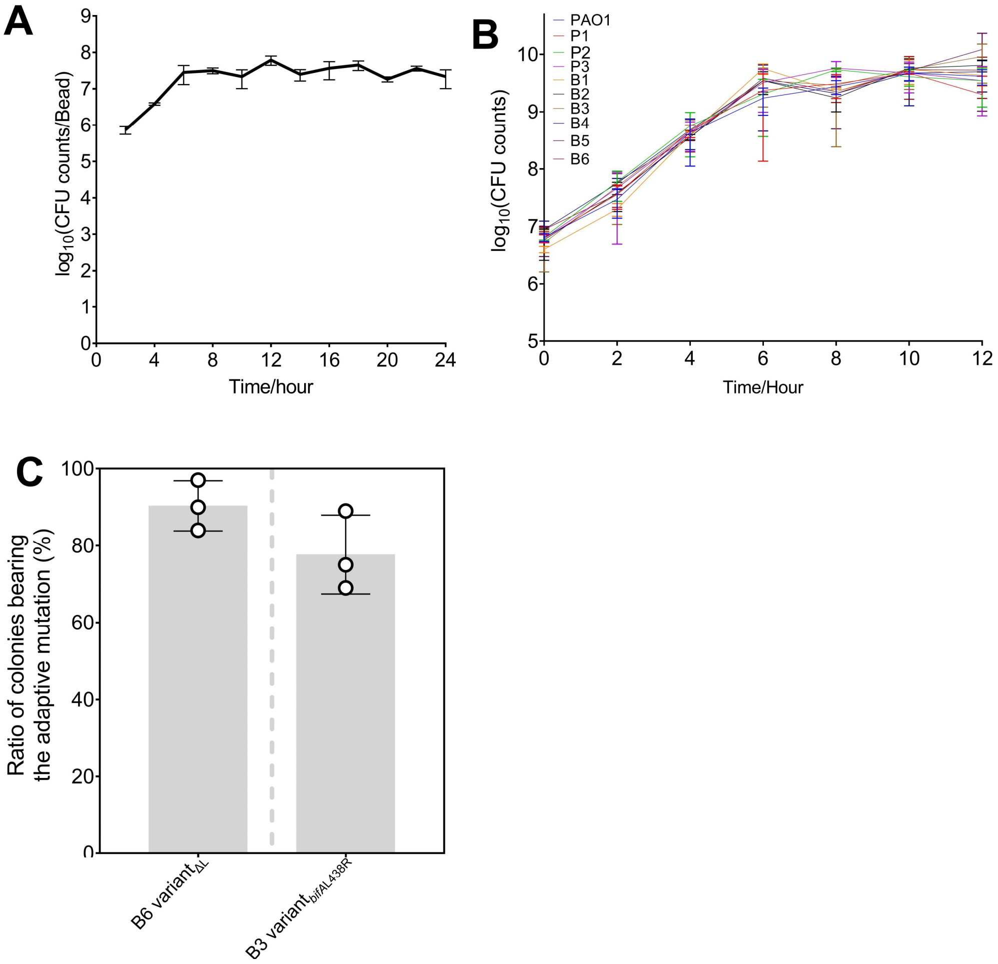

Supplementary Fig. 1: Two fitness-adaptative mutations emerged in the experimental evolution of *Pseudomonas aeruginosa* PAO1 biofilm. **A** PAO1 biofilm biomass was measured by numeration of colony formation units (CFU) during the 24 hours of growth. **B** Growth curves of *P. aeruginosa* PAO1 and its derived populations. **C** Ratio of colonies bearing the non-synonymous *bifA*L438R in the B3 population and the Pf4 large deletion in the B6 population.

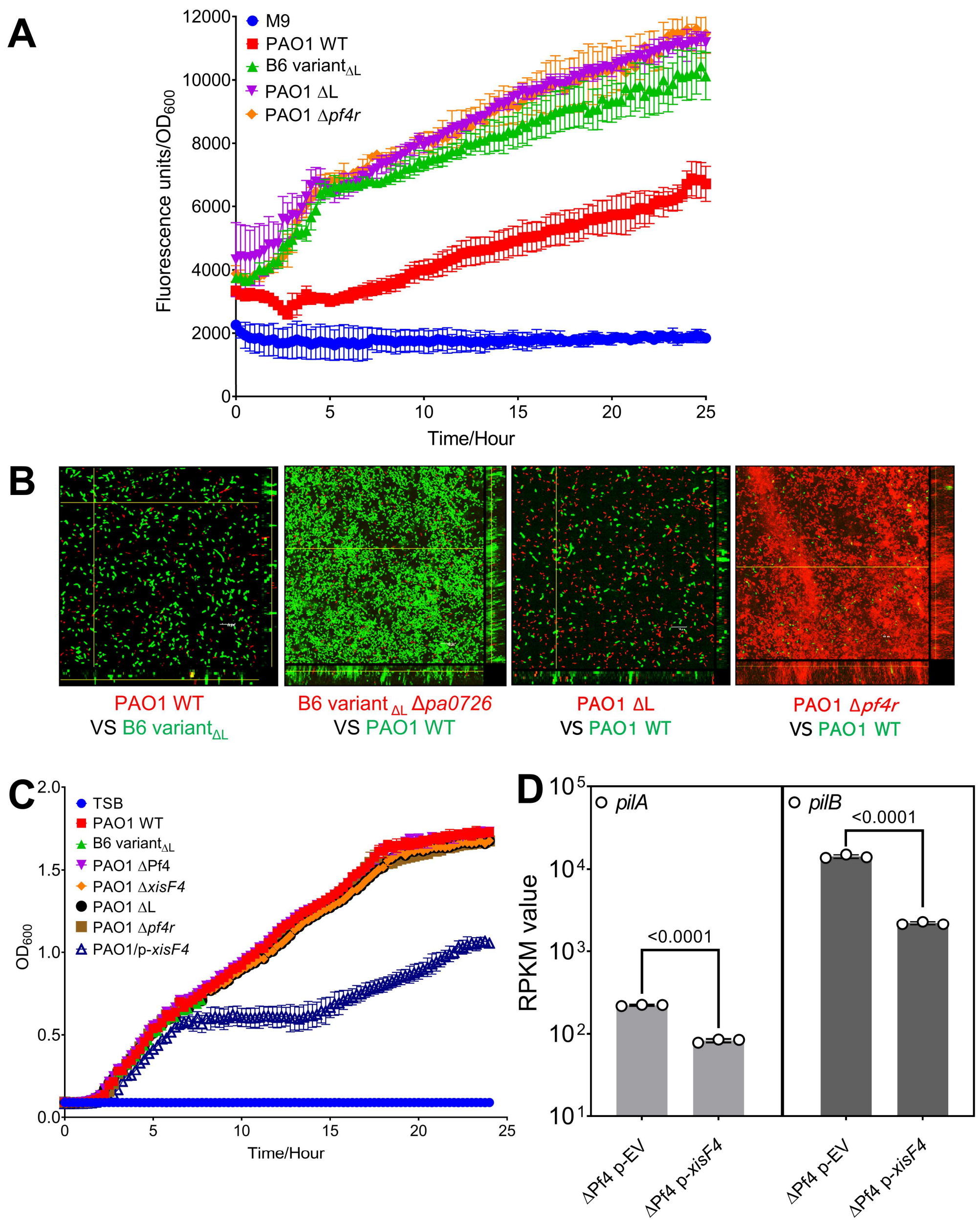

Supplementary Fig. 2: A large deletion in the Pf4 prophage region caused Pf4 superinfection and a concomitant resistance to Pf4, thus conferring the fitness adaptation to the evolved clone B6 variant$_{\Delta L}$ in biofilms. (A) *xisF4* gene expression dynamics in various strains were detected using the P*xisF4-gfp* reporter system. (B) Biofilm formed by mixed strains at 24 hpi imaged by confocal laser scanning microscope. Bacterial fluorophore was indicated by the font color below the images. (C) Growth curves of *P. aeruginosa* PAO1, its derived mutants and evolved clones. (D) Effect of *xisF4* overexpression on the transcription of two T4P-associated genes, *pilA* and *pilB*. RPKM, Reads Per Kilobase per Million mapped reads. *P* values were determined by two-tailed unpaired Student's *t*-test. Each experiment was repeated at least three times.

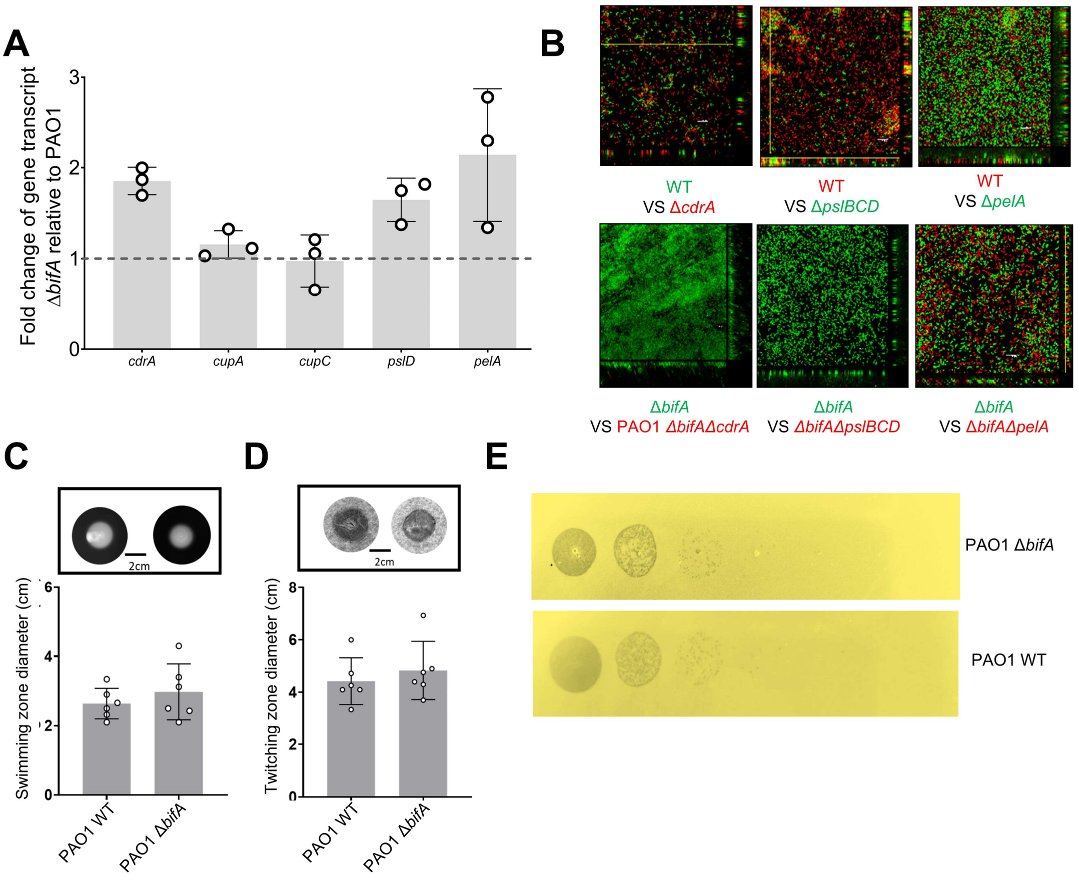

Supplementary Fig. 3: A non-synonymous mutation in *bifA* conferred a biofilm competition advantage to a *P. aeruginosa* PAO1 evolved derivative, whereas the Pel exopolysaccharide, type IV pili, flagellum and Pf4 phages were not involved. (A) Relative transcript levels *of cdrA, cupA1, cupC1, pslD*, and *pelA* in the Δ*bifA* mutant normalized against the PAO1 WT. (B) Biofilm morphology of mixed strains imaged by confocal laser scanning microscope. Bacterial fluorophores were indicated by the font color. (C) Swimming and (D) twitching motility of PAO1 WT and PAO1 Δ*bifA* after 24 hours of incubation, respectively. (E) PFUs formed by phage lysates from PAO1 and the isogeneic Δ*bifA* mutant after 12 hours of incubation. Each experiment was conducted at least three times. Values correspond to means ± standard deviations. Statistical significance was determined using two-tailed unpaired *t*-test or one-way ANOVA with Tukey post-hoc tests using a 95% confidence interval.

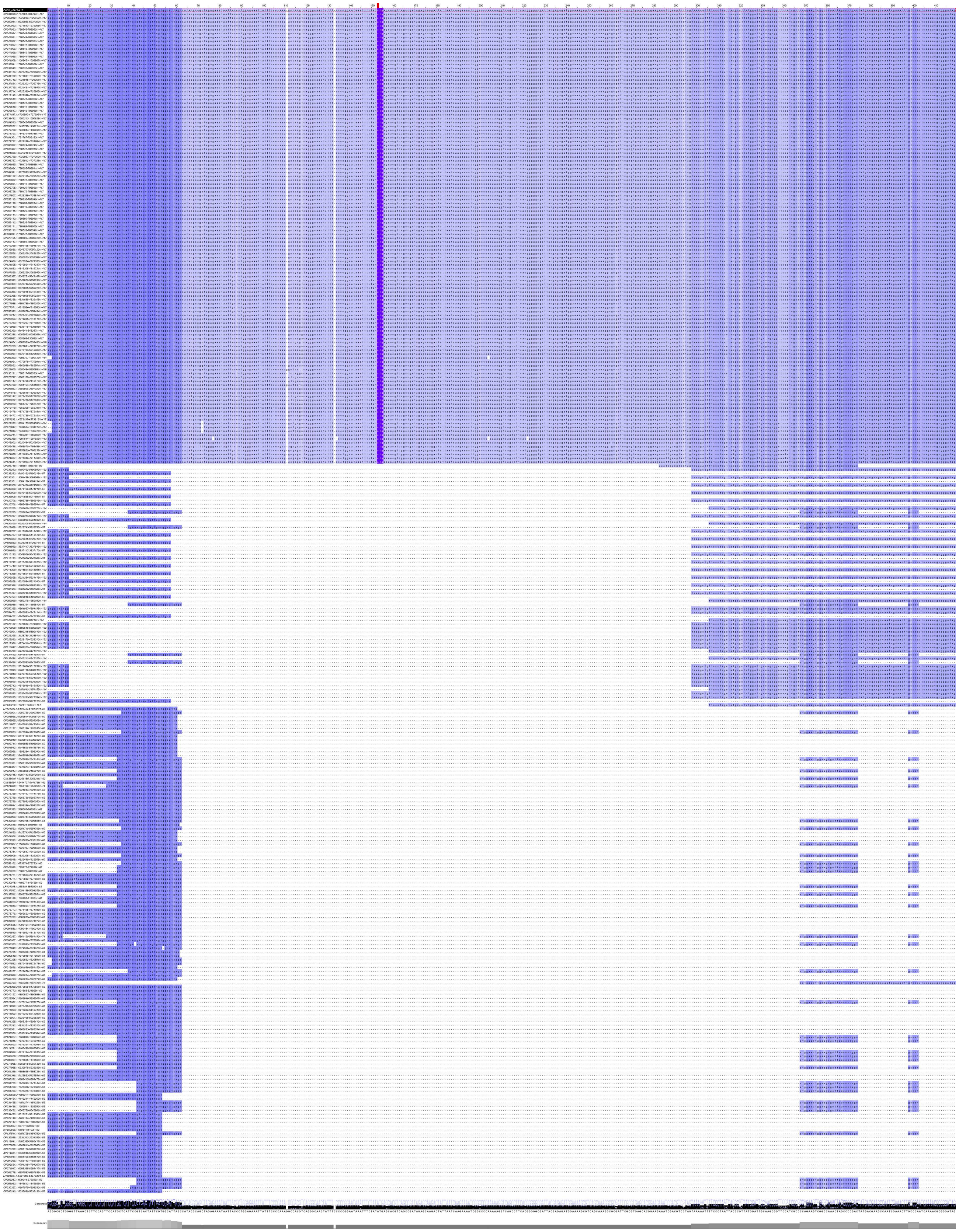

Supplementary Fig. 4: Gene sequence alignment of *pf4r*. The most similar *pf4r* homologues from 500 Pf prophages were aligned with PAO1 *pf4r* gene sequence using the MMseqs2. The consensus sequence was shown below the sequence alignment. The ATG start codon in the open reading frame that was clearly defined in a previous study was marked with dark purple [1].

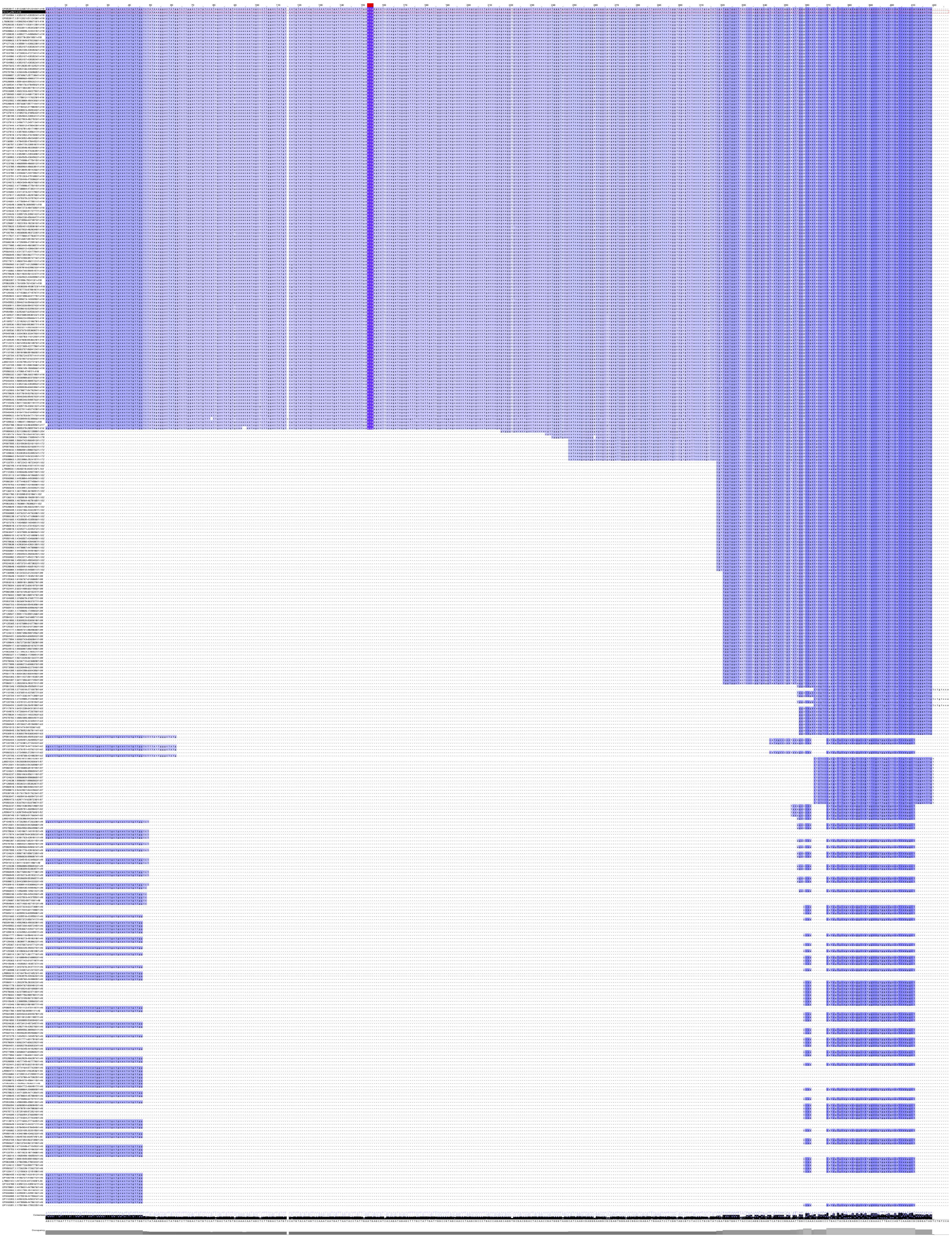

Supplementary Fig. 5: Gene sequence alignment of *pf5r*. The most similar *pf5r* homologues from 500 Pf prophages were aligned with PA14 *pf5r* using the MMseqs2. The consensus sequence was shown below the sequence alignment. The ATG start codon was marked with dark purple.

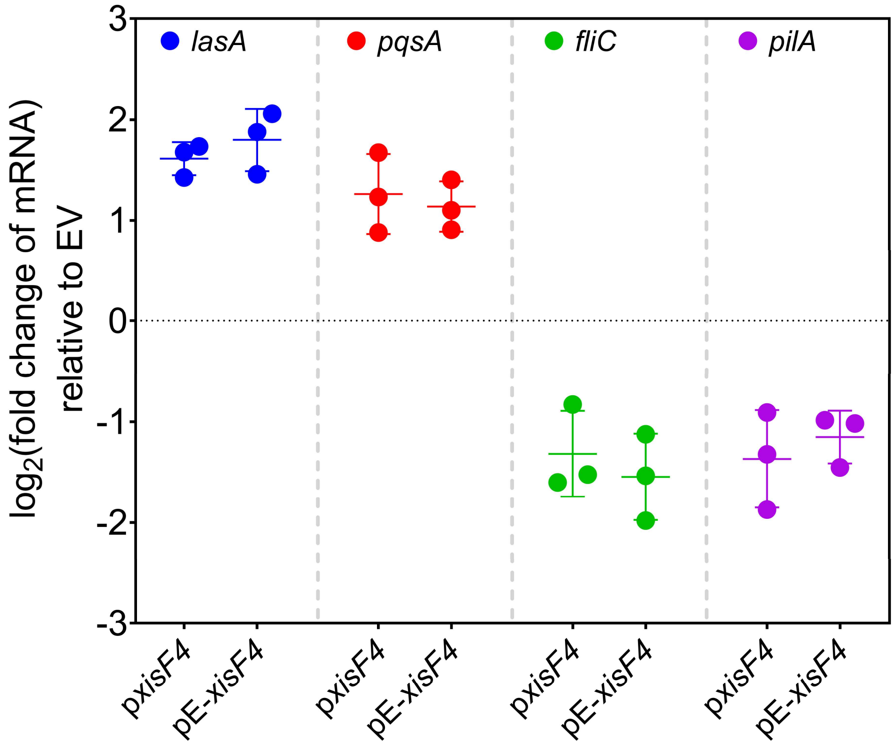

Supplementary Fig. 6: Expression fold changes of four representative virulence genes detected by RT-qPCR in PAO1 Δ*xisF4* with either *xisF4* or E-*xisF4* induced with 2 M arabinose under the control of an inducible promoter pBAD. EV, empty control.

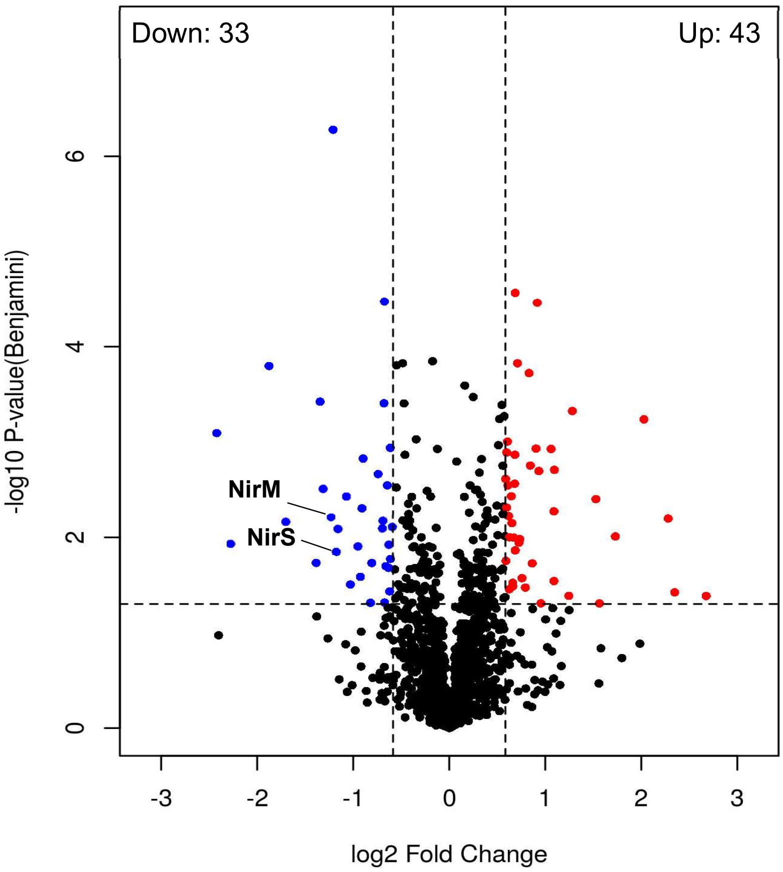

Supplementary Fig. 7: Volcano plot showing the differentially expressed proteins in evolved clone B3 variant$_{\textit{bifA}\mathrm{L438R}}$ relative to PAO1. Proteomic comparison analysis revealed that NirS and NirM were downregulated due to *bifA* inactivation. Specific proteins, NirM and NirS, were marked.

Supplementary methods

Pf4 replicative-form (RF) quantification

The numbers of Pf4 RF molecules were quantified using primers (Pf4-Cf/Cr) flanking closely the phage attachment site (*attP*) (Table S2). The value of frequency of RF number were normalized by reference gene (*gyrB*), which is a single-copy housekeeping gene indicating the number of total chromosomes. Strains were grown overnight and adjusted to an OD_{600} of 0.05 in LB. Cells were collected at OD_{600} ~ 1.0 by centrifugation (12000 rpm for 1 min), and DNA was extracted using DNA Isolation Kit (TIANGEN, PRC). Carbenicillin and 10 mM arabinose were supplemented into the medium for strains carrying pHERD20^{T}-based plasmids from an OD_{600}~0.05 to overexpress the target genes. Total DNA (50-200 ng) was then used as the template for the qPCR reaction using TB Green® Premix Ex Taq™ II (Takara, Japan).

RT-qPCR and RNA-seq

Strains were grown overnight and adjusted to an OD_{600} of 0.05 in LB. Cells at the log phase (OD_{600}~0.6) were collected for RNA extraction using EASYspin Plus RNA Mini kit (Aidlab, PRC). The medium was supplemented with carbenicillin for strains carrying pHERD20^{T}-based plasmids, and 10 mM arabinose was added at OD_{600} ~0.1 for 4 hours. The cDNA synthesis was conducted using PrimeScript™ RT Master Mix (Takara, Japan). Total cDNA (100 ng) was used for qRT-PCR using the TB Green® Premix Ex Taq™ II FAST qPCR (Takara, Japan). The level of the *gyrB* gene transcript was used to normalize the gene expression data. For transcriptomic analysis, RNA was sequenced on an Illumina Hiseq Xten platform and 150 bp paired-end reads were generated. The quality of raw sequence data was assessed using FastQC (Babraham Bioinformatics). Adaptor sequences were removed by trimming function in CLC. RNA sequence analysis was done using “RNA-seq analysis’ module in CLC genomics Workbench 20 (CLC Bio, Denmark) using PAO1 reference genome downloaded from NCBI database. Differential gene expression was analyzed using DESeq2 package in R software. Differentially expressed genes are defined when the adjusted *P* values is less than 0.05 and the (log_2[fold change] is greater than 1 or less than -1.

Proteomic analysis

P. aeruginosa strains were grown in TSB for 6 hours at 37°C. To harvest the bacteria, the cultures were centrifuged at 5000 rpm for 10 min at 4°C and transferred to a new 2 mL screw-cap tube. The

RIPA buffer (Beyotime, China) and glass beads were added, followed lysed by a cell disruption device at 70 Hz for 3 min. The proteins were reduced with 10 mM DTT (Sigma-Aldrich, USA) for 1 hour at 70°C, then the alkylation was carried out using 50 mM iodoacetamide (IAA, Sigma-Aldrich) for 15 min at room temperature in the dark. The samples were desalted, and the buffer was changed to 0.5 M ammonium bicarbonate buffer. Then the proteins were digested with trypsin (Promega, USA). Peptides were separated on a C18 tip column (75 μm × 250 mm, Acclaim PepMap RSLC, 2 μm) and analyzed with a Q Exactive Plus mass spectrometer (Thermo Fisher Scientific Inc., USA). The data were compared with the Uniprot reference proteome of *P. aeruginosa* (strain ATCC 15692) reference proteome database. Differentially expressed proteins are defined when the adjusted *P* values is greater than 0.05 and the fold change is more than 1.5 or less than -1.5.

Protease assay

Total extracellular proteolytic activity of *P. aeruginosa* strains was evaluated using skim milk agar plates as previously described [2]. Fresh overnight cultures in LB were rinsed and adjusted to an OD_{600} of 0.01, and 2 μL was spotted on the skim milk agar plates (25% (v/v) LB, 4% (w/v) skim milk, 1.5% (w/v) agar) followed by incubation at 37°C for 8 h. The diameter of transparent zones reflecting extracellular proteolytic activity of *P. aeruginosa* strains was quantified from captured photographs.

Bioinformatics analysis

The phylogenetic analysis of BifA among *P. aeruginosa* strains in the nr database was conducted using the online BLASTP tool of Pseudomonas Genome Database with an e-value threshold of 0.001, a coverage of 70% and specifying the organism as "*Pseudomonas aeruginosa*." Subsequently, for a more detailed mutation analysis of the EAL and GGDEF domains, BifA amino acid sequence of PAO1 was aligned against 7482 homologous sequences using the 'search' option in the MMseqs2 package with default parameters [3]. Phylogenetic trees were then constructed by performing multiple sequence alignment analysis using MAFFT v7.505 [4] and applying neighbor-joining method through Fasttree [5]. For the investigation of PA0724 (minor coat protein) homologues, we used MMseq2 to search in the database of 7982 *P. aeruginosa* strains for PA0724 utilizing the 'search' option in the MMseqs2 package with a coverage threshold of 80% and a minimal amino acid identity of 40% [3], and we used Seqkit to extract the sequences [6], in

which the PA0724 gene was located at the center and flanked by upstream and downstream regions with each being around 6000 bp in length to ensure the inclusion of potential prophage region. CheckV, a widely used approach to perform phage gene detection on the target sequences [7], was used to exclude the sequences without other prophage genes. Consequently, a pool of 2645 eligible Pf prophages, among which the presence/absence of excisionases and phage repressors were obtained for further analysis. The phylogenetic tree of PA0724 homologues was constructed using the same method as we described above. To assess the conservation of the *pf4r* and *pf5r* genes, 279 *pf4r* sequences and 244 *pf5r* sequences (including the promoter and coding regions) were obtained using the online BLASTP tool of NCBI with an e-value of 0.001. Subsequently, multiple sequence alignments were performed using MAFFT v7.505 [4], which were then visualized using Jalview [8]. To analyze the distribution of Pf4r and Pf5r among *P. aeruginosa* strains, the amino acid sequences of Pf4r from PAO1 and Pf5r from PA14 were aligned against the 2645 *P. aeruginosa* genomes using the 'search' option in the MMseqs2 package with a coverage threshold of 80% and a minimal amino acid identity of 40% [3]. All phylogenetic trees were displayed and annotated using the iTOL online tool [9].

Biofilm biomass and morphology observation

Biofilm formation in 96-well microtiter plates was quantified as previously described [10]. 100 μL of bacterial cultures diluted by TSB to OD_{600} of 0.01 was seeded in the wells. Biofilm were incubated at 37°C for 24 h, followed by washing with sterilized ultrapure water and treatment with 0.3% crystal violet (CV). Next, it was washed again with ultrapure water and resuspended in 30% acetic acid. The relative biofilm biomass was quantified by measuring the optical density of the CV staining at 550 nm, using a microplate reader (Tecan, Switzerland). For 3D imaging of biofilm morphology, fresh bacterial cultures at OD_{600} of 0.01 were seeded into a cell culture dish inlaying a glass coverslip (World Precision Instruments, USA). Liquid culture was aspirated off after 24 h of incubation at 37°C, and the biofilm was gently rinsed twice with 200 mL sterile PBS to remove the free-living bacteria. Confocal images were acquired using a CLSM (OLYMPUS, Japan) with a 100× oil immersion objective.

Plaque assay

Plaque assays were performed as previously described [1, 11]. Bacterial lawns were prepared by mixing 1 ml of bacterial stationary culture OD_{600}~4 was mixed with 4 mL of LB-top agar (8 g L^{-1}

agar, 0.1% glucose, 5 mM $CaCl_2$) at 40°C and rapidly poured over 5 ml of LB-bottom medium (10 g L^{-1} agar, 0.1% glucose, 5 mM $CaCl_2$). Phages in filtered supernatants were serially diluted 10-fold in LB and spotted onto lawns of the indicated indicator strain. Plaques were imaged after 18 h of growth at 37°C.

Motility assays

As previously described [12], twitching motility was assessed by stab inoculating the indicated strains at OD_{600}~0.2 through a 1.2% LB agar to the plastic dish bottom. After 24 h of incubation, the agar was removed, and the zone of motility on dish bottom was measured after staining with 0.05% Coomassie brilliant blue. For swarming assay, a fresh overnight culture in LB of each strain was adjusted to an OD_{600} of 1, and 2 μL was spotted onto the middle of the swarming agar containing 8 g L^{-1} nutrient broth (Oxoid, United Kingdom), 5 g L^{-1} glucose (Sangon, PRC), and 0.5% agar (Fisher Scientific). For the swimming motility, a colony from the fresh overnight culture on LB agar was stabbed onto the surface of agar with a sterile toothpick on the swimming agar containing 25 g L^{-1} LB and 0.3% agar. Agar was supplemented with 10 mM arabinose and carbenicillin when to overexpress target genes.

Alpha2 model of protein structure

Protein structure predictions were performed using the https://www.uniprot.org/online site [13, 14]. UniProt entries were found by using the peptide search function of the query peptide sequences. Some regions with low pLDDT may be unstructured. We then mapped the amino acid residue positions of the XisF4 or BifA structure models using the structure tool of the online site.

Protein expression and purification

The coding region of *xisF4* was cloned into plasmid pET28a (Table S5) using primers listed in Table S6. The genes were then expressed in *E. coli* BL21(DE3). The strains were grown to OD_{600} of 0.6, and then 0.4 mM isopropyl β-D-1-thiogalactopyranoside (IPTG, Sigma, USA) was used to induce gene expression at 16°C for 24 h to obtain XisF4. The His6-fusion proteins were purified from 400 mL *E. coli* BL21(DE3) carrying the pET28a plasmid derivatives using Ni-NTA resin (Sangon Biotech, Shanghai, PRC). Protein purity was assessed by sodium dodecyl sulfate-polyacrylamide gel electrophoresis (SDS-PAGE). Finally, the concentration of purified proteins was determined by BCA protein assay kit (Sangon Biotech, PRC).

Electrophoretic mobility shift assay (EMSA)

The promoter regions of *pa0727* (229 bp), *pilA* (243 bp), *lasA* (283 bp) and *narL* (190 bp) were PCR-amplified from PAO1 using the corresponding primers listed in Table S6. DNA fragments (40 μg) were purified using the TIANgel Mini Purification Kit (TIANGEN, PRC), mixed with the purified proteins and incubated at room temperature for 1 h. Then the binding reaction samples were run on a 6% DNA retardation gel at 100 V in 0.5 × TBE.

Transcriptional reporter assays

For the *cdrA, rsmY, rsmZ* promoter-reporter fusion strains, the plasmid P*cdrA-gfp,* P*rsmY-gfp* and P*rsmZ-gfp* was introduced into *Pseudomonas* strains by electroporation. Transformants were selected on LB agar plates containing carbenicillin. Overnight cultures were adjusted using fresh ABTGC medium, and 100 μL of dilute cultures at OD_{600} ~ 1.0 were transferred to a polystyrene black 96-well plate (Corning). Cells were measured on a Spark plate reader (Tecan) for OD_{600} and GFP fluorescence. Fluorescence was measured as fluorescence intensity units (FIU) using an excitation of 488 nm and emission of 535 nm. The FIU values were normalized to OD_{600}, and the average autofluorescence from the empty vector control was subtracted for each strain. Three biological replicates, each with three technical replicates, were performed.

Pyocyanin measurement

Pyocyanin production measurement was performed as described previously [15]. Briefly, overnight cultures were diluted to an OD_{600} of 1.0, and 50 μL was used to inoculate 5 mL of LB. After 18 h, 3 mL of chloroform was added to 5 mL of culture supernatant, and the tube was vortexed twice for 5 min. The tubes were centrifuged at 12,000 rpm for 5 min, and the bottom phase was transferred to a new tube containing 1 mL of 0.2 N HCl. The tubes were vortexed twice for 5 min each time and centrifuged at 7,000 rpm for 2 min. The OD_{520} of the top phase was measured.

Cell culture and cytotoxicity assay

The cytotoxicity of the ancestor, mutants and evolved isolates were measured by using human lung epithelium-like A549 cells, an adenocarcinoma cell line. A549 cells were purchased from ATCC and cultured in Dulbecco's modified Eagle medium (DMEM) supplemented with 10% fetal bovine serum (GIBCO, USA), 100 U mL^{-1} penicillin, and 100 μg mL^{-1} streptomycin at 37°C and 5% CO_2. A549 cells were then seeded in 24-well plates, washed with sterile phosphate buffered saline (PBS) twice and resuspended in DMEM medium devoid of FBS. A549 cells were infected

with bacteria at a Multiplicity of Infection (MOI) of 10 at 37°C in 5% CO_2 incubator. After 6 h infection, the culture supernatants were collected for detection of lactate dehydrogenase (LDH) activities using commercially LDH cytotoxicity kit (YAESEN Bio, PRC) according to standard procedure.

***In vivo* infection of *Galleria mellonella* larvae**

Galleria mellonella larvae infection was performed as previously described [16]. The larvae were purchased from Huiyude Biotechnology Co. (Tianjin, China), with five weeks old post hatching, approximately 2 cm in length and 5 grams in weight. Overnight bacterial cultures were diluted to 1×10^3 CFU mL^{-1} and injected into the leftmost foreleg of larvae using a 10-μL syringe (Anting Microsampler Factory, PRC). The injected larvae were incubated at 37°C, and survival rates were monitored.

References for Supplementary materials

1. Li Y, Liu X, Tang K *et al*. Excisionase in Pf filamentous prophage controls lysis-lysogeny decision-making in *Pseudomonas aeruginosa.* Mol Microbiol. 2019; **111**: 495-513. https://doi.org/10.1038/ncomms5462
2. Cai W, Liao H, Lu M *et al*. New evolutionary insights into RpoA: A novel quorum sensing reprograming factor in *Pseudomonas aeruginosa.* Mol Biol Evol. 2023; **40**: msad203. https://doi.org/10.1093/molbev/msad203
3. Steinegger M, Söding J. MMseqs2 enables sensitive protein sequence searching for the analysis of massive data sets. Nat Biotechnol. 2017; **35**: 1026-8. 1028. https://doi.org/10.1038/nbt.3988
4. Katoh K, Standley DM. MAFFT multiple sequence alignment software version 7: Improvements in performance and usability. Mol Biol Evol. 2013; **30**: 772-80. https://doi.org/10.1093/molbev/mst010
5. Price MN, Dehal PS, Arkin AP. FastTree: Computing large minimum evolution trees with profiles instead of a distance matrix. Mol Biol Evol. 2009; **26**: 1641-50. https://doi.org/10.1093/molbev/msp077
6. Shen W, Le S, Li Y, *et al*. SeqKit: A cross-platform and ultrafast toolkit for FASTA/Q file manipulation. Plos One. 2016; **11**: e0163962. https://doi.org/10.1371/journal.pone.0163962
7. Nayfach S, Camargo AP, Schulz F, *et al*. CheckV assesses the quality and completeness of metagenome-assembled viral genomes. Nat Biotechnol. 2021; **39**: 578-85. https://doi.org/10.1038/s41587-020-00774-7
8. Waterhouse AM, Procter JB, Martin DMA et al. Jalview Version 2-a multiple sequence alignment editor and analysis workbench. Bioinformatics. 2009; **25**: 1189-91. https://doi.org/10.1093/bioinformatics/btp033
9. Letunic I, Bork P. Interactive Tree Of Life (iTOL) v5: an online tool for phylogenetic tree display and annotation. Nucleic Acids Res. 2021; **49**: W293-W6. https://doi.org/10.1093/nar/gkab301
10. Tang M, Liao S, Qu J et al. Evaluating bacterial pathogenesis using a model of human airway

organoids infected with Pseudomonas aeruginosa biofilms. Microbiol Spectr. 2022; **10**: e02408-22. https://doi.org/10.1128/spectrum.02408-22

11. Cai YM, Yu KW, Liu JH et al. The c-di-GMP phosphodiesterase PipA (PA0285) regulates autoaggregation and Pf4 bacteriophage production in Pseudomonas aeruginosa PAO1. Appl Environ Microbiol. 2022; **88**: e0003922. https://doi.org/10.1128/aem.00039-22
12. Schmidt AK, Fitzpatrick AD, Schwartzkopf CM et al. A filamentous bacteriophage protein inhibits type IV pili to prevent superinfection of Pseudomonas aeruginosa. mBio. 2022; **13**: e02441-21. https://doi.org/10.1128/aem.00039-22
13. Coudert E, Gehant S, de Castro E et al. Annotation of biologically relevant ligands in UniProtKB using ChEBI. Bioinformatics. 2022; **39**(1): btac793. https://doi.org/10.1093/bioinformatics/btac793
14. Consortium TU. UniProt: the universal protein knowledgebase in 2023. Nucleic Acids Res. 2022; **51**: D523-D31. https://doi.org/10.1093/nar/gkac1052
15. Essar DW, Eberly L, Ayele et al. Identification and characterization of genes for a second anthranilate synthase in Pseudomonas aeruginosa: interchangeability of the two anthranilate synthases and evolutionary implications. J Bacteriol. 1990; **172**: 884 -900. https://doi.org/10.1128/jb.172.2.884-900.1990
16. Seed KD, Dennis JJ. Development of Galleria mellonella as an alternative infection model for the Burkholderia cepacia complex. Infect Immun. 2008; **76**: 1267-75. https://doi.org/10.1128/IAI.01249-07
